# Mitigating vertical crystallization inhomogeneity in blade-coated Sn–Pb perovskites for flexible all-perovskite tandem modules

**DOI:** 10.1039/d6el00168h

**Published:** 2026-10-23

**Authors:** Jincheng Luo, Jonathan S. Austin, Amber Wright, Chih-Jen Shih, Fan Fu

**Affiliations:** a Laboratory for Thin Films and Photovoltaics, Empa – Swiss Federal Laboratories for Materials Science and Technology Dübendorf Switzerland fan.fu@empa.ch; b Institute for Chemical and Bioengineering, ETH Zürich Zürich Switzerland

## Abstract

Flexible all-perovskite tandem photovoltaics combine high power-conversion efficiency (PCE) and compatibility with lightweight, conformable substrates, making them attractive for aerospace systems and portable electronics. Gas-quenching-assisted meniscus-based coating offers a promising route towards scalable, high-throughput manufacturing. However, its limited solvent-extraction capability hinders the formation of thick, high-quality mixed Sn–Pb perovskite absorbers required for efficient tandem devices. Here, we identify pronounced vertical morphological and optoelectronic inhomogeneity in blade-coated mixed Sn–Pb perovskite films, with more defects in the upper region, arising from a thorough-thickness drying gradient. To address this limitation, we introduce the additive d-homoserine lactone hydrochloride (d-HLH) into the precursor ink to regulate perovskite crystallization. d-HLH promotes larger grains and suppresses nonradiative recombination, particularly in the upper region, thereby improving vertical homogeneity. Consequently, flexible mixed Sn–Pb perovskite solar cells achieve a PCE of 18.4% with improved operational stability. Integration with a blade-coated wide-bandgap top subcell yields flexible monolithic all-perovskite tandem solar cells with a champion PCE of 23.6% and a *T*_80_ lifetime of 273 h under maximum-power-point tracking at ∼55 °C. Flexible tandem mini-modules further deliver a PCE of 20.6% with a stabilized efficiency of 19.8% over an aperture area of 10.8 cm^2^. This work reveals an overlooked limitation in gas-quenched mixed Sn–Pb perovskites and provides a crystallization-regulation strategy for scalable, high-performance flexible all-perovskite tandem photovoltaics.

## Abstract 2

d-HLH-modulated crystallization improves quality and mitigates vertical heterogeneity in gas-quenched, blade-coated mixed Sn–Pb perovskites, enabling flexible all-perovskite tandem solar mini-modules with efficiencies above 20%.

## Graphical abstract

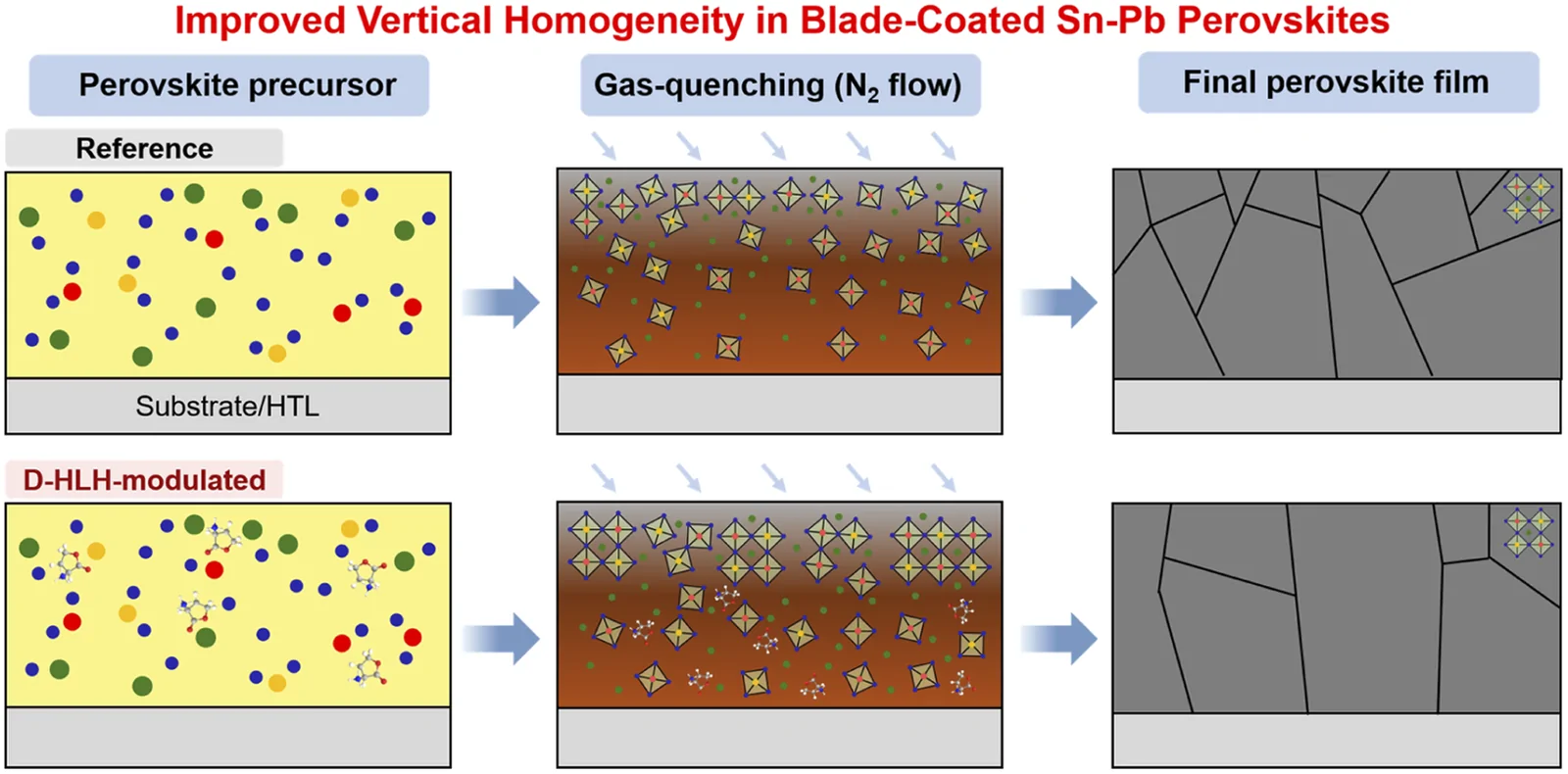

**Broader context**

Expanding solar power beyond conventional rigid panels requires efficient photovoltaic technologies compatible with lightweight, conformable substrates. Flexible all-perovskite tandem solar modules offer the potential for high power per unit weight, creating opportunities for aerospace systems, portable electronics and other integrated photovoltaic applications. To enable these opportunities, gas-quenching-assisted meniscus-based coating offers a promising route to scaling up flexible all-perovskite tandems. However, limited capability of solvent extraction can cause uneven drying and crystallization across thick mixed Sn–Pb perovskite films, compromising absorber quality and device performances. Here, we introduce an additive into the precursor ink to modulate crystallization and improve through-thickness homogeneity during blade coating. This strategy promotes grain growth, reduces nonradiative recombination and improves the efficiency and operational stability of flexible mixed Sn–Pb perovskite solar cells. Flexible all-perovskite tandem mini-modules deliver a stabilized efficiency of 19.8% over an aperture area of 10.8 cm^2^. These findings highlight the importance of controlling through-thickness crystallization of mixed Sn–Pb perovskites and develop a feasible strategy to advance its scalable fabrication for efficient flexible tandem solar modules.

## Introduction

Metal halide perovskites offer tunable bandgaps across ∼1.2–3.0 eV through compositional adjustment, making them particularly suitable for tandem and multi-junction devices.^1–4^ By integrating a wide-bandgap (WBG) mixed-halide perovskite top subcell with a narrow-bandgap (NBG) mixed Sn–Pb perovskite bottom subcell through an interconnecting layer, all-perovskite tandems can achieve power-conversion efficiencies (PCEs) beyond those attainable with single-junction devices, owing to broadened absorption ranges and reduced thermalization losses.^4,5^ The low-temperature solution processability of perovskites also allows their deposition on lightweight, flexible substrates using cost-effective and energy-efficient manufacturing techniques, such as meniscus-based coating methods, which are compatible with high-throughput roll-to-roll (R2R) production.^6,7^ Flexible all-perovskite tandems are therefore particularly attractive for aerospace systems, portable electronics, and other integrated photovoltaic applications, in which a high power-to-weight ratio is critical.^8,9^

Flexible all-perovskite tandem solar cells (TSCs) have advanced rapidly in recent years, reaching a certified PCE of 28.0%.^10^ Nevertheless, progress towards scalable flexible tandems remains extremely limited.^11–13^ The highest reported flexible all-perovskite tandem module achieved a certified PCE of 23.0% over an aperture area of ∼20 cm^2^.^11^ Meniscus-based deposition methods, including doctor-blade and slot-die coating, are well suitable for upscaling because they can support continuous processing and efficient ink utilization in R2R systems.^14,15^ Gas quenching is similarly attractive for high-throughput R2R manufacturing because it enables inline solvent removal without antisolvent application or vacuum-assisted quenching.^16^

However, producing thick, high-quality perovskite absorbers by gas-quenching-assisted blade coating remains challenging due to limited solvent-extraction capacity.^17,18^ Efficient tandem devices commonly require NBG absorbers approaching 1 µm in thickness to provide sufficient near-infrared absorption and achieve current matching.^19,20^ The difficulty of obtaining high-quality mixed Sn–Pb perovskite films across large-area flexible substrates therefore represents a critical obstacle to efficient tandem modules. Volatile cosolvents and heated-gas quenching have been investigated to accelerate solvent removal and improve the quality of thick mixed Sn–Pb films.^21,22^ Moreover, additive engineering has been well developed and widely employed to effectively control the crystallization process of spin-coated mixed Sn–Pb perovskites.^23,24^ Representative strategies include thiocyanate-based additives that coordinate with metal-halide precursors, multifunctional ligands that stabilize the intermediate phase, and Sn-selective binding agents that retard crystallization of the Sn-containing component, thereby promoting synchronized crystallization for high-quality mixed Sn–Pb perovskite films.^25–27^ However, corresponding strategies for gas-quenched blade-coated mixed Sn–Pb perovskite films remain poorly explored. Developing additives for regulation of through-thickness crystallization is therefore essential for producing homogeneous, high-quality NBG films on large-area flexible substrates.

Here, we identified pronounced vertical inhomogeneity in blade-coated flexible mixed Sn–Pb perovskite films. The upper region contained smaller grains and exhibited stronger nonradiative recombination than the region near the buried interface. We associate this vertical heterogeneity with the through-thickness supersaturation gradient generated during gas quenching. To address this limitation, d-homoserine lactone hydrochloride (d-HLH) was introduced into the mixed Sn–Pb perovskite precursor ink. d-HLH regulated the crystallization of mixed Sn–Pb perovskite films, resulting in enlarged perovskite grains and enhanced photoluminescence emission, with reduced vertical heterogeneity. As a result, the d-HLH-containing flexible mixed Sn–Pb perovskite solar cells (PSCs) achieved a champion PCE of 18.4%, compared with 16.9% for the reference cells. When integrated with a WBG perovskite top subcell, flexible all-perovskite TSCs achieved the highest efficiency over 23.6%. Flexible all-perovskite tandem solar mini-modules further achieved a PCE of 20.6%. Overall, this work uncovers a quality-limiting factor in gas-quenched flexible mixed Sn–Pb perovskite absorbers and demonstrates an effective crystallization-regulation strategy for scalable, high-performance flexible all-perovskite tandem photovoltaics.

## Results and discussion

### Vertical heterogeneity in blade-coated mixed Sn–Pb perovskites

In this work, FA_0.7_MA_0.3_Pb_0.5_Sn_0.5_I_3_ (FA = formamidinium; MA = methylammonium) was selected as the NBG (∼1.26 eV) perovskite composition. Doctor-blade coating was used to deposit spatially uniform wet films, and subsequent gas quenching promoted solvent extraction and established the supersaturation required for nucleation and grain growth during thermal annealing. The mixed Sn–Pb precursors were dissolved in DMF/DMSO, in which DMSO strongly coordinates with Sn^2+^ and Pb^2+^ to form Lewis acid–base adducts and regulate the crystallization process of mixed Sn–Pb perovskite films.^25,28^ Because gas quenching extracts strongly coordinating DMSO less efficiently than antisolvent treatment, blade-coating inks typically contain a lower DMSO fraction of approximately 10%, compared with approximately 25% in spin-coating ink.^17,21,29^ Gas quenching can also produce a through-thickness drying gradient, especially in the thick films required for efficient mixed Sn–Pb PSCs.^30^ An appropriate gas-quenching pressure should therefore be optimized to balance the surface overdrying against excessive residual solvent near the buried interface, thereby promoting more favorable crystallization for dense, high-quality mixed Sn–Pb perovskite films.^30,31^ We optimized the gas-quenching pressure to 2.0 bar, at which the flexible mixed Sn–Pb PSCs achieved their best performance (Fig. S1).

The rapid gas quenching process can create a surface-to-substrate drying and supersaturation gradient because solvent removal occurs at the gas-facing surface, while the solvent and precursor redistribution inside the film occurs by diffusion. This gradient can become significant when evaporation is faster than internal diffusion, especially for relatively thick, viscous, or strongly coordinating precursor solutions. Previous studies on metal halide perovskites have reported surface-first drying or crystallization, together with residual solvent and buried-interface voids after gas-assisted coating.^30,32^ These observations support the expected supersaturation gradient, consequently causing a difference in nucleation density from the top surface to the buried interface (Fig. 1a). The more rapidly dried upper region contains a higher concentration of nuclei than the region near the buried interface, which is unfavorable for vertically homogeneous crystallization. During subsequent thermal annealing, the higher nucleation density near the top surface is expected to produce smaller grains, whereas fewer nuclei near the buried interface allows the growth of larger grains. Cross-sectional scanning electron microscope (SEM) images of mixed Sn–Pb perovskite films on a rigid glass/indium tin oxide (ITO) substrate confirmed this grain-size difference (Fig. S2). Such a vertically inhomogeneous crystallization process can lead to the formation of numerous small perovskite grains and therefore a high density of grain boundaries, which may introduce abundant defect states.

**Fig. 1:**
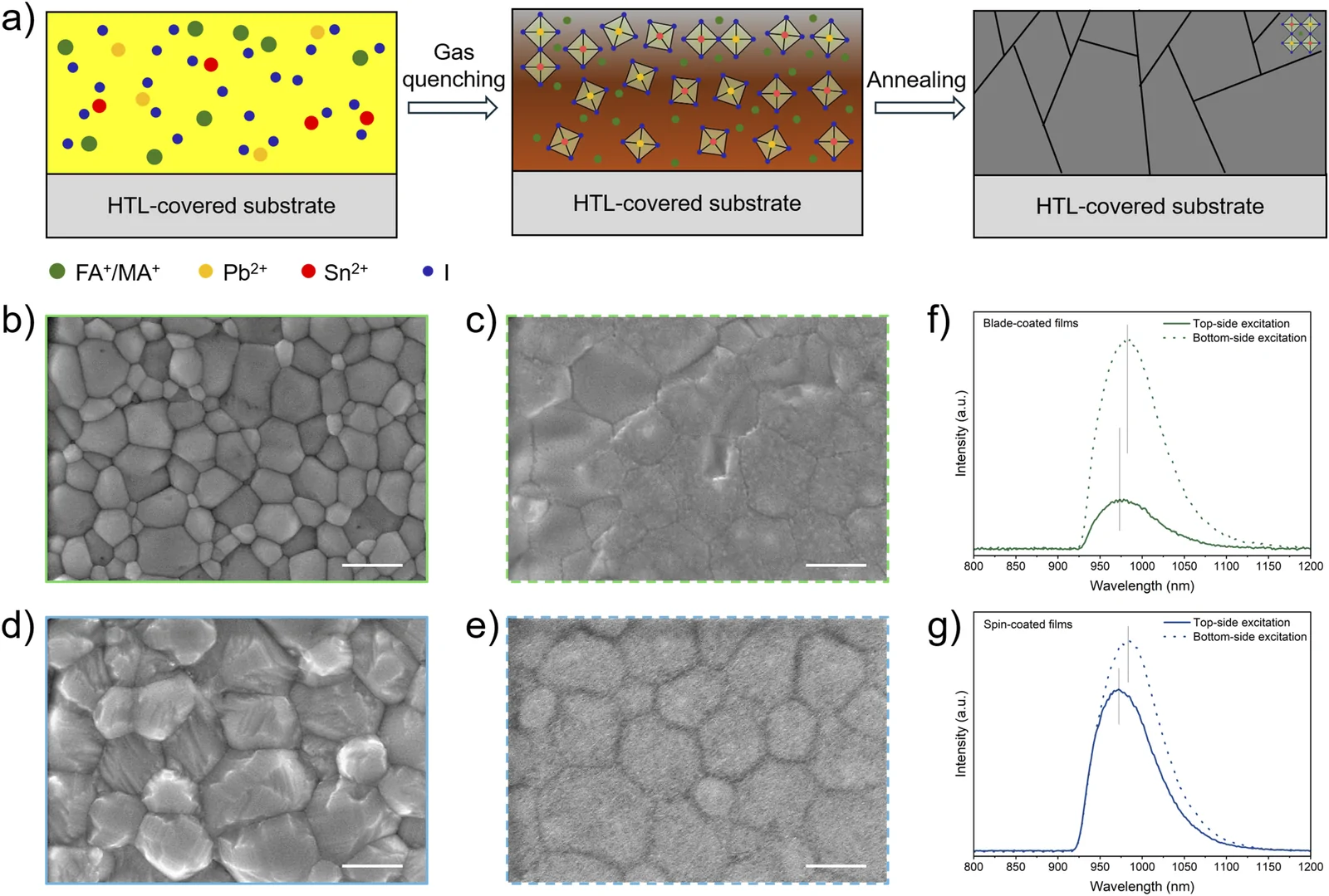
Through-thickness morphology and photoluminescence of flexible mixed Sn–Pb perovskite films. (a) Schematic illustration of the nucleation and crystallization processes of mixed Sn–Pb perovskite films through gas-quenching-assisted doctor-blade coating and subsequent thermal annealing. (b–e) Top-view SEM images of mixed Sn–Pb perovskite films, deposited by (b and c) gas-quenching-assisted blade coating and (d and e) antisolvent-quenching-assisted spin coating, acquired from the (b and d) top surface and (c and e) buried interface. Scale bar: 500 nm. (f and g) Steady-state PL spectra of (f) blade-coated and (g) spin-coated mixed Sn–Pb perovskite films, measured under excitation from either the top-surface (top) or buried-interface (bottom) side.

SEM measurements were performed to compare top-surface and buried-interface morphologies of flexible mixed Sn–Pb perovskite films prepared by blade coating with gas quenching and spin coating with antisolvent quenching. Judging from exposed grain surfaces, the blade-coated films exhibited substantially smaller grains near the top surface than near the buried interface (Fig. 1b and c), consistent with the crystallization mechanism discussed above. In contrast, the spin-coated films showed comparable grain sizes at the top surface and buried interface (Fig. 1d and e). This distinct grain-size gradient supports our interpretation that gas quenching produces a vertical gradient of supersaturation and nucleation density, causing nonuniform crystallization across the thick perovskite layer. These processes account for the smaller grains near the top surface, impeding the formation of high-quality mixed Sn–Pb perovskite films.

Steady-state photoluminescence (PL) spectroscopy was performed to investigate how vertical variation in grain size affects the optoelectronic quality of mixed Sn–Pb perovskite films. The films were excited with a 655 nm laser from either the top surface or the buried interface, thereby preferentially probing the region near the illuminated side. Excitation from the buried interface produced substantially stronger PL emission than excitation from the top surface (Fig. 1f). The weaker top-side emission is consistent with the greater density of grain boundaries and enhanced trap-assisted nonradiative recombination in the finer-grained upper region.^33^ Top-side excitation also produced a blue-shifted PL peak relative to bottom-side excitation. Since we observed no significant vertical compositional gradient of Sn/Pb throughout the film (Fig. S3), this shift may be associated with a Burstein–Moss effect arising from stronger unintentional p-type doping,^34^ although other factors such as depth-dependent strain and carrier redistribution may also contribute to this observed PL peak shift.^35^ By contrast, the spin-coated mixed Sn–Pb perovskite film displayed a much smaller difference in PL intensity between top-side and bottom-side excitations (Fig. 1g). Collectively, these results reveal pronounced vertical heterogeneity and a more defective upper region in gas-quenching-assisted blade-coated mixed Sn–Pb films, which constrain the performance of mixed Sn–Pb PSCs and all-perovskite tandems.

### Additive-modulated crystallization regulation and improved optoelectronic properties

To improve the quality of mixed Sn–Pb perovskite, especially in the upper region of the films, we introduced the additive d-HLH into the precursor ink. Fig. S4 shows its chemical structure, featuring a cyclized lactone ring with carbonyl and protonated amino groups. These functionalities are expected to interact with perovskite components through metal coordination (–C=O⋯Pb^2+^/Sn^2+^), hydrogen bonding, and electrostatic interactions, influencing the formation of perovskite nuclei and regulating the crystallization process of mixed Sn–Pb perovskite films (Fig. 2a). Previous spin-coating studies with higher DMSO content inks have revealed those expected interactions between d-HLH and multiple perovskite precursor components, which contribute to regulating the perovskite crystallization process; however we note the strength of these interactions may differ in this gas-quenched system with lower DMSO content ink.^26,36,37^ Specifically, d-HLH promotes the conversion of perovskite-solvent intermediates into perovskite nuclei while moderating the subsequent crystal growth, thereby facilitating the formation of high-quality perovskite films. Hereafter, samples prepared without and with d-HLH are denoted as reference and d-HLH, respectively. The d-HLH addition has negligible influence on the absorbance and optical bandgap of mixed Sn–Pb perovskite films (Fig. S5), indicating that it does not alter the bulk composition and electronic structure of mixed Sn–Pb perovskites.

**Fig. 2:**
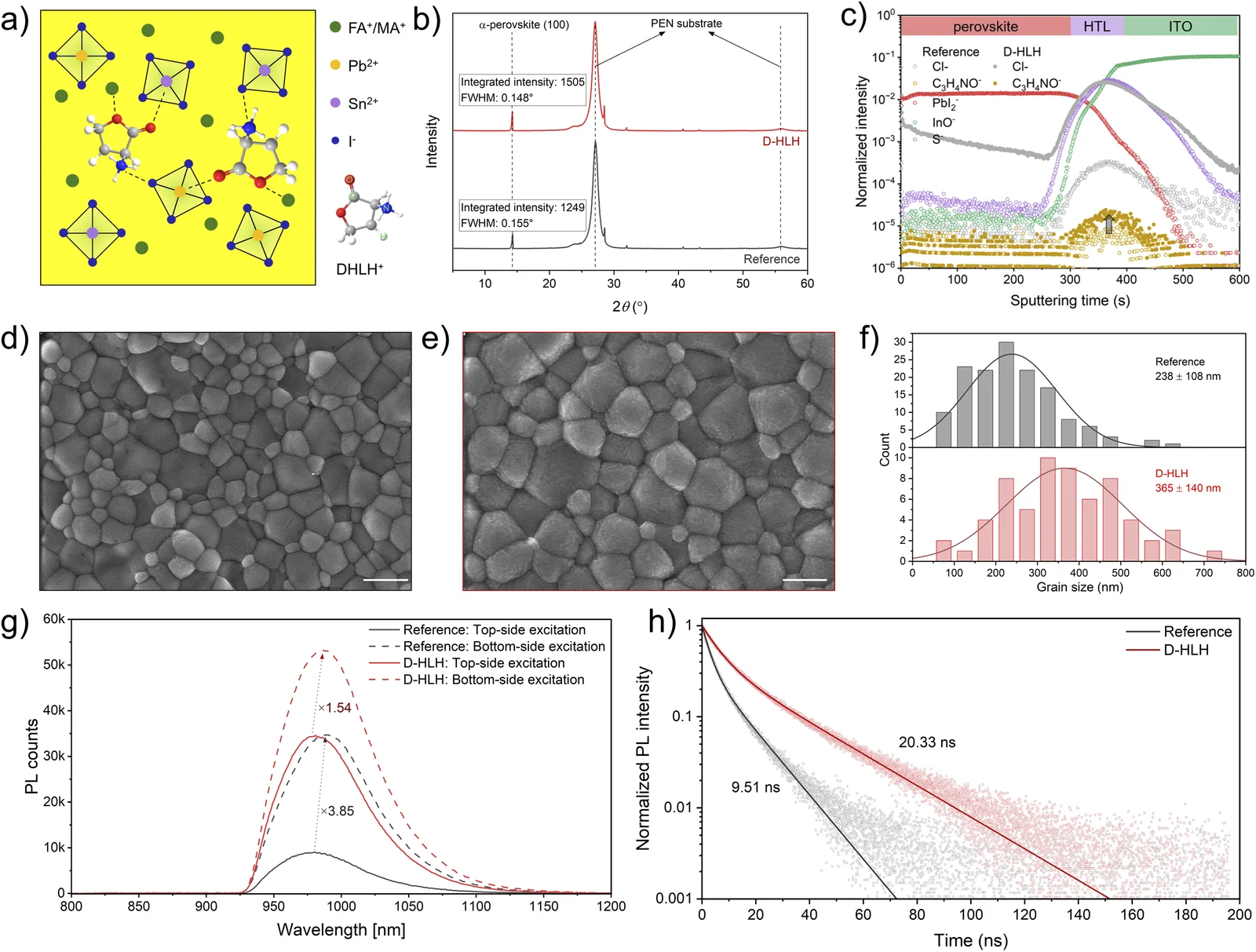
d-HLH-modulated regulation of crystallization, morphology, and optoelectronic properties in flexible mixed Sn–Pb perovskite films. (a) Schematic diagram of interaction between d-HLH^+^ and perovskite components. (b) XRD patterns of reference and d-HLH flexible mixed Sn–Pb perovskite films. (c) ToF-SIMS depth profile of Cl^−^, PbI_2_^−^, InO^−^, S^−^, and C_3_H_4_NO^−^ in mixed Sn–Pb perovskite films. (d and e) Top-view SEM images of (d) reference and (e) d-HLH mixed Sn–Pb perovskite films. Scale bar: 500 nm. (f) Corresponding top-surface grain-size distributions extracted from the SEM images. (g) Steady-state PL spectra of reference and d-HLH mixed Sn–Pb perovskite films under excitation from either the top-surface or buried-interface side. Each spectrum represents the average of five measurements collected at 5 different positions across a 5 × 5 cm^2^ substrate. (h) TRPL decays and biexponential fitting curves of reference and d-HLH mixed Sn–Pb perovskite films.

We first examined the influence of d-HLH on the early-stage crystallization of blade-coated flexible mixed Sn–Pb perovskite films using X-ray diffraction (XRD). Measurements were performed on gas-quenched mixed Sn–Pb perovskite films without thermal annealing (Fig. S6). Both reference and d-HLH films showed diffraction peaks from the photoactive α-phase perovskite, whereas no crystalline perovskite–solvent intermediate phase was observed. Notably, the quenched d-HLH film exhibited a stronger and narrower perovskite (100) diffraction peak than the reference film. Its full width at half maximum (FWHM) decreased from 0.168° to 0.157°, indicating greater structural coherence within the as-quenched perovskite domains. During thermal annealing, these perovskite domains coalesced and grew, forming more crystalline perovskite grains. In the annealed films, the integrated intensity of the perovskite (100) diffraction peak at ∼14.26° increased after d-HLH addition, while its FWHM decreased from 0.155° to 0.148° (Fig. 2b). These results suggest that d-HLH regulates early-stage crystallization, and this early structural advantage carries through to yield greater crystallinity after thermal annealing.

Next, we investigated the vertical distribution of d-HLH^+^ and Cl^−^ ions using time-of-flight secondary ion mass spectrometry (ToF-SIMS). The C_3_H_4_NO^−^ fragment was selected as a characteristic marker for d-HLH^+^ in the perovskite films (Fig. S7). Depth profiles revealed that d-HLH^+^ was concentrated predominantly near the buried interface of mixed Sn–Pb perovskite films (Fig. 2c). Cl^−^ was enriched at both interfaces, and some ions were present throughout the bulk of the film. Based on these observations, we propose that, as crystallization of gas-quenched perovskite films can proceed predominantly from the free surface toward the substrate,^32,38^ the advancing crystallization front progressively excludes the relatively bulky d-HLH^+^ species from the crystallizing perovskite domains, leading to their accumulation toward the buried interface. By contrast, the smaller Cl^−^ may be less strongly excluded during crystallization and thus partially remain in bulk.^39^

The influence of d-HLH on crystallization was further reflected in the final film morphology. Top-view SEM images revealed significantly larger apparent grains in the d-HLH film than in the reference film (Fig. 2d and e). Quantitative analysis showed that the average grain size increased by approximately 53%, from 238 to 365 nm (Fig. 2f and S8). By comparison, buried-interface SEM images showed less pronounced morphological differences between the two films (Fig. S9). Complementary cross-sectional SEM images of rigid mixed Sn–Pb perovskite films further showed that d-HLH addition reduced the population of small grains in the upper region and promoted the formation of more columnar grains extending through the film thickness (Fig. S10). These observations suggest that d-HLH affects grain development more strongly in the upper region than near the buried interface. We attribute this spatial dependence to the through-thickness solvent-extraction gradient generated during gas quenching. Rapid solvent removal at the exposed surface produces earlier supersaturation and nucleation in the upper region, where d-HLH-modulated regulation of nucleus formation and subsequent coarsening may consequently be most pronounced.

Overall, the additive d-HLH regulates the early-stage crystallization process of gas-quenched mixed Sn–Pb perovskites. This regulation produces structurally more coherent as-quenched domains, greater crystallinity after annealing, and enlarged grains, particularly in the upper region of the film. The preferential improvement of the lower-quality upper region mitigates the vertical heterogeneity in flexible mixed Sn–Pb perovskite films prepared by gas-quenching-assisted blade coating. The reduced grain-boundary density is expected to reduce grain-boundary-associated nonradiative recombination, thereby potentially improving the optoelectronic properties of mixed Sn–Pb perovskite films.^33^

To examine the effect of d-HLH on the optoelectronic properties of mixed Sn–Pb perovskite films, we performed steady-state and time-resolved PL (TRPL) measurements to evaluate charge-carrier recombination dynamics within both reference and d-HLH mixed Sn–Pb perovskite films. For steady-state PL measurements, the excitation was applied from either the top-surface or buried-interface side. Because the above-bandgap excitation is absorbed predominantly near the illuminated surface, the two configurations preferentially weigh the PL response toward the top and buried regions, respectively.^40^ The d-HLH-containing film exhibited enhanced PL intensity under both excitation geometries (Fig. 2g and S11), indicating suppressed nonradiative recombination throughout the entire perovskite absorber. Notably, the PL enhancement was more pronounced under top-surface excitation than under buried-interface excitation. This difference suggests that d-HLH produced a stronger optoelectronic improvement in the upper region, consistent with the more pronounced morphological changes observed in Fig. 2d and e. This preferential improvement of the initially lower-quality upper region reduces the optoelectronic disparity across the film thickness, leading to improved vertical homogeneity. We note that the absolute PL intensities measured under the two excitation geometries can also be affected by differences in reflectance, parasitic absorption by the PEN/ITO/HTL stack, and self-absorption of the emitted PL. Quantitative analysis of the experimentally accessible optical losses, including reflectance and parasitic absorption, indicates that these effects alone cannot account for the pronounced bottom/top PL intensity ratio and its substantial reduction from 3.85 ± 0.53 to 1.54 ± 0.17 upon d-HLH incorporation (Note S1).

TRPL was measured under pulsed excitation from the top-surface side to preferentially generate carriers away from the perovskite/poly(3,4-ethylenedioxythiophene):polystyrene sulfonate (PEDOT:PSS) interface, thereby minimizing interfacial charge-transfer effects. Fig. 2h shows the TRPL decays of reference and d-HLH mixed Sn–Pb perovskite films with biexponential fitting curves. The average PL lifetime increased from 9.51 ns for the reference film to 20.33 ns for the d-HLH-containing film (Table S1). This prolonged lifetime is consistent with the suppressed nonradiative recombination and agrees with the enhanced steady-state PL intensity. Complementary transient photovoltage (TPV) measurements showed that the effective photovoltage decay time increased from 260 to 391 µs after d-HLH addition (Fig. S13), further indicating suppressed device-level carrier recombination.^41^

### Photovoltaic performance of flexible mixed Sn–Pb perovskite solar cells and mini-modules

Flexible NBG PSCs were fabricated on 5 × 5 cm^2^ substrates with the configuration of polyethylene naphthalate (PEN)/ITO/PEDOT:PSS/mixed Sn–Pb perovskite/C_60_/bathocuproine (BCP)/Cu. We first investigated the concentration-dependent effect of d-HLH on the photovoltaic performance of blade-coated flexible mixed Sn–Pb PSCs (Fig. S14, S15 and Table S2). The optimal concentration was 1 mol%, at which the PCEs increased from 13.6% ± 0.3% for reference cells to 15.2% ± 0.3%. This improvement arose from simultaneous increases in the open-circuit voltage (*V*_OC_), short-circuit current density (*J*_SC_), and fill factor (FF), consistent with the enhanced crystallinity and enlarged grain sizes of d-HLH-containing films. The d-HLH device also exhibited a lower dark current density, supporting reduced carrier recombination and increased *V*_OC_ (Fig. S16).

Light-intensity-dependent *J*–*V* measurements were subsequently performed to examine the device recombination behavior. Analysis of *V*_OC_ as a function of light intensity yielded ideality factors of 1.41 and 1.11 for the reference and d-HLH devices, respectively (Fig. S17a). This shift toward unity indicates a reduced contribution of trap-assisted recombination,^42^ in agreement with the enhanced PL emission. This interpretation is further supported by the higher recombination resistance extracted from electrochemical impedance spectroscopy (Fig. S17b).

We subsequently applied a blade-coated ethylenediammonium diiodide (EDAI_2_) post-deposition treatment (PDT) to improve the charge-carrier dynamics at the perovskite/C_60_ interface.^43,44^ The synergistic effect of EDAI_2_ PDT and d-HLH further boosts flexible mixed Sn–Pb PSCs to higher efficiencies, primarily through improvements in the *V*_OC_ and FF (Fig. 3a and S18). The best-performing d-HLH device achieved a PCE of 18.4% under reverse voltage scan, with a *V*_OC_ of 0.82 V, a *J*_SC_ of 28.49 mA cm^−2^, and an FF of 79.0%, compared with the reference device, which achieved a PCE of 16.9%, a *V*_OC_ of 0.78 V, a *J*_SC_ of 28.13 mA cm^−2^, and an FF of 77.1% (Fig. 3b and Table S3). The integrated *J*_SC_ values derived from external quantum efficiency (EQE) spectra are 28.24 mA cm^−2^ and 27.74 mA cm^−2^ for the d-HLH and reference devices, respectively. These values agree well with the *J*_SC_ values obtained from *J*–*V* measurements (Fig. 3c). Power tracking at fixed bias for 100 s yielded stabilized PCEs of 17.8% and 15.9% for d-HLH and reference devices, respectively (Fig. S19). Moreover, d-HLH mixed Sn–Pb PSCs fabricated on a single 5 × 5 cm^2^ PEN/ITO substrate exhibited good spatial uniformity in PCE, with a relative standard deviation of 3.1% across 16 cells (Fig. S20).

**Fig. 3:**
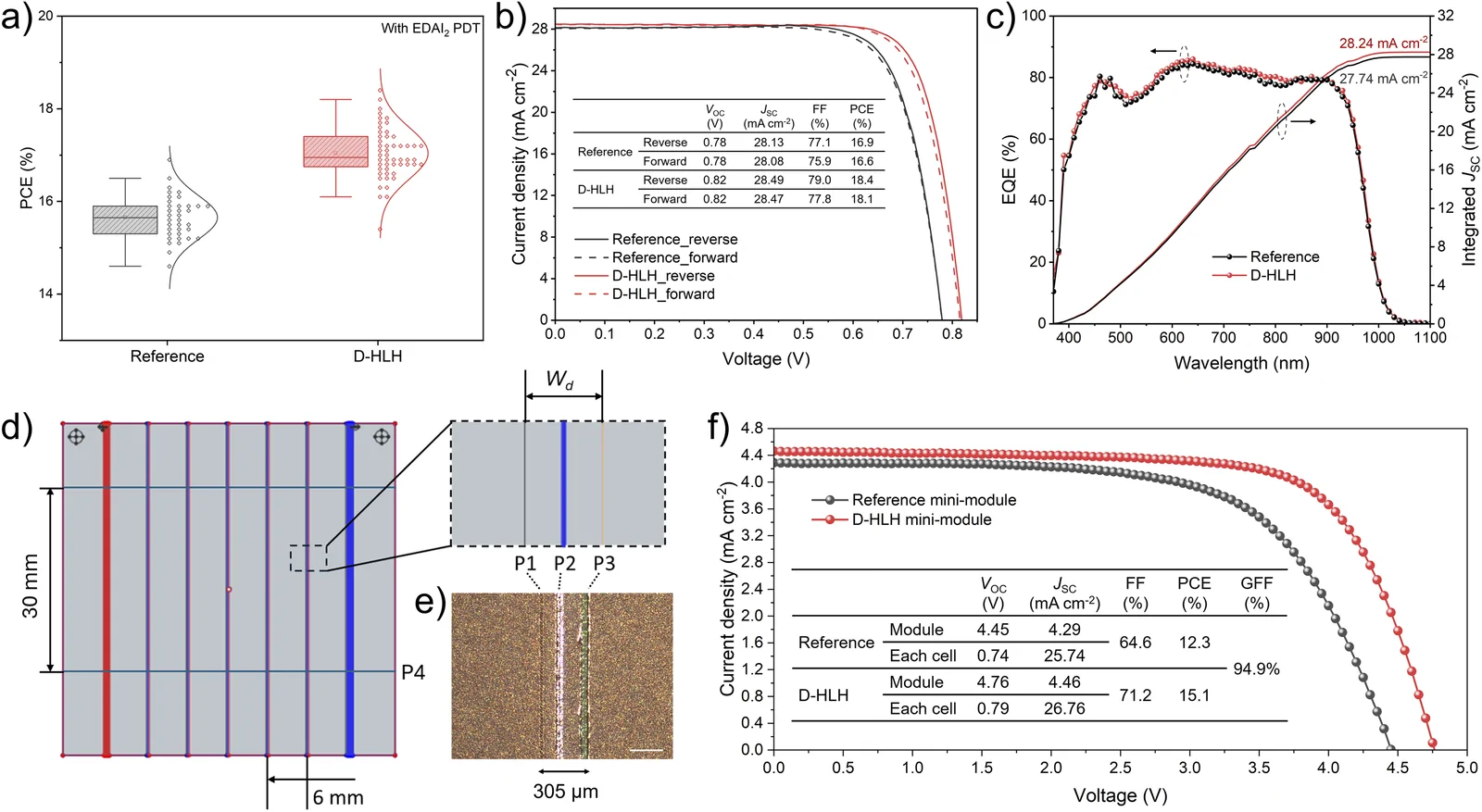
Photovoltaic performance of flexible mixed Sn–Pb perovskite solar cells and mini-modules. (a) Statistical PCE distributions of reference and d-HLH flexible mixed Sn–Pb PSCs after EDAI_2_ PDT, including 36 reference and 52 d-HLH cells from multiple batches. (b) *J*–*V* characteristics and (c) EQE spectra of the best-performing reference and d-HLH PSCs. (d) Laser-scribing layout used to fabricate flexible mixed Sn–Pb perovskite solar mini-modules. (e) Optical microscope image of a flexible mixed Sn–Pb perovskite solar mini-module near the dead zone. Scale bar: 200 µm. (f) *J*–*V* characteristics of the best-performing reference and d-HLH flexible mixed Sn–Pb perovskite solar mini-modules under reverse voltage scan.

Beyond photovoltaic efficiency, we also evaluated the operational stability of flexible mixed Sn–Pb PSCs under continuous one-sun-equivalent LED illumination. The devices were operated at their maximum-power point (MPP) and heated by illumination to ∼55 °C. The encapsulated reference devices dropped to less than 50% of their peak PCEs after 40 h (Fig. S21). By contrast, the encapsulated d-HLH devices exhibited much improved stability, with over 60% of their peak PCEs remaining after 140 h. This improvement may be partly associated with enhanced morphology and crystallinity and reduced grain boundary density of the d-HLH-containing mixed Sn–Pb perovskite films, which can reduce defect-mediated recombination and susceptible pathways for grain-boundary-associated degradation.^25,45^

Flexible mixed Sn–Pb perovskite solar mini-modules were further fabricated using a conventional P1–P2–P3 laser-scribing strategy, with the layout shown in Fig. 3d. Extra P4 scribe lines defined an aperture area of 10.8 cm^2^, comprising six series-connected cells, each 6 mm wide and 3 cm long. The dead-zone width (*W*_d_) was limited to approximately 305 µm, corresponding to a geometric fill factor (GFF) of 94.9% (Fig. 3e). Fig. S22 shows a photograph of an encapsulated flexible mini-module. The d-HLH-containing flexible mixed Sn–Pb perovskite solar mini-module achieved a PCE of 15.1%, with a *V*_OC_ of 4.76 V, a *J*_SC_ of 4.46 mA cm^−2^, and an FF of 71.2% (Fig. 3f). By comparison, the reference mini-module achieved a PCE of 12.3%. Stabilized power output measurements yielded PCEs of 14.1% and 11.3% for the d-HLH and reference mini-modules, respectively (Fig. S23).

### Photovoltaic performance of flexible all-perovskite tandem solar cells and mini-modules

We further fabricated monolithic flexible all-perovskite TSCs with a configuration of PEN/ITO/nickel oxide (NiO_*x*_)/(4-(3,6-dimethyl-9*H*-carbazol-9-yl)butyl)phosphonic acid (Me-4PACz)/WBG (FA_0.8_Cs_0.2_PbI_1.8_Br_1.2_, 1.8 eV) perovskite/C_60_/tin oxide (SnO_*x*_)/Au cluster (1.2 nm)/PEDOT:PSS/NBG perovskite/C_60_/BCP/Cu, as shown in Fig. 4a. The detailed fabrication methods and corresponding performance of flexible WBG PSCs have been discussed in our previous work.^12^

**Fig. 4:**
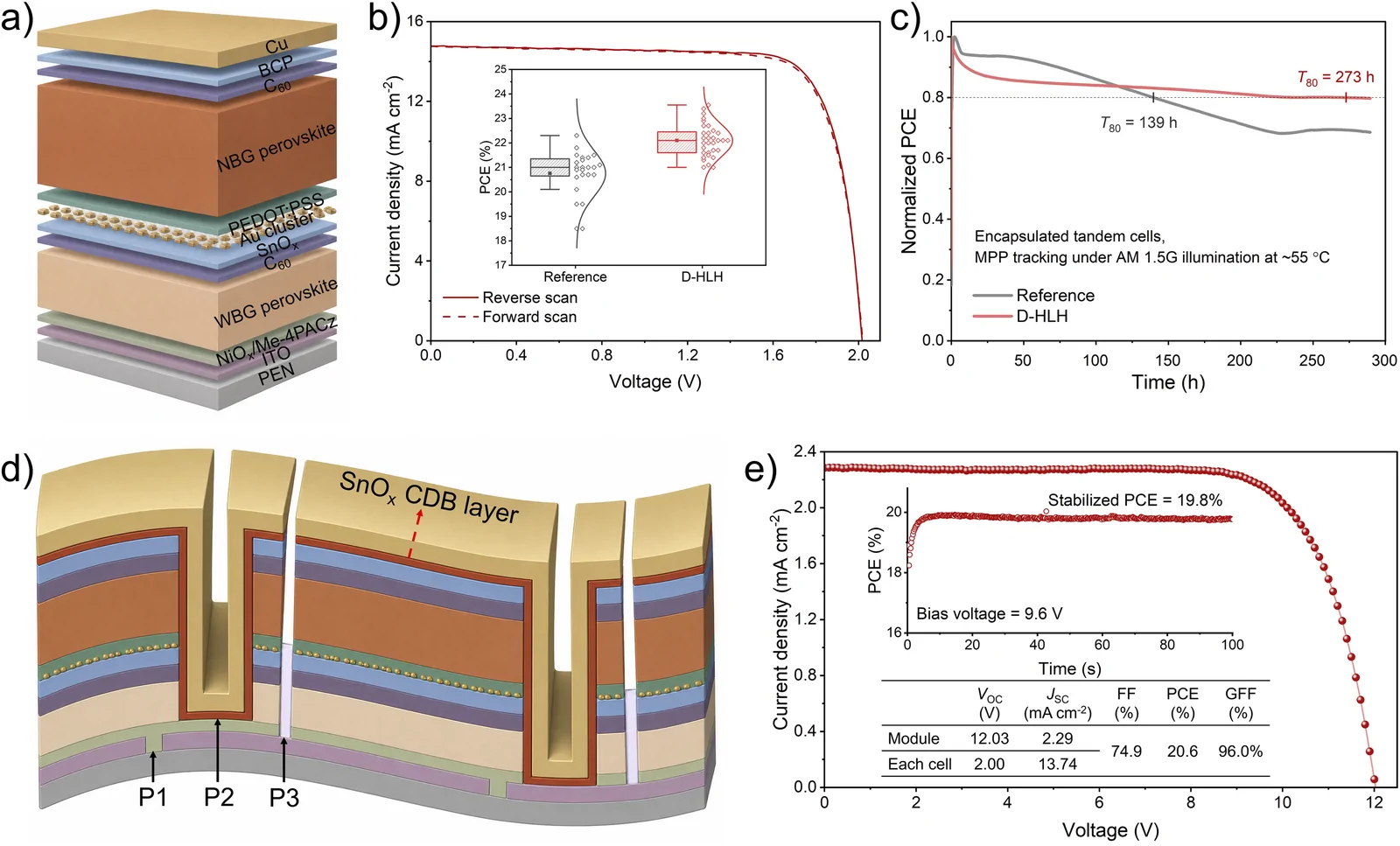
Photovoltaic performance of flexible all-perovskite tandem solar cells and mini-modules. (a) Schematic configuration of a flexible all-perovskite TSC. (b) *J*–*V* characteristics of the best-performing flexible all-perovskite TSC. The inset compares the PCE distributions of 24 reference and 32 d-HLH flexible tandem cells from multiple batches. (c) Operational stability of reference and d-HLH flexible tandem cells assessed by MPP tracking under continuous 1 sun illumination at ∼55 °C. (d) Schematic configuration of a flexible all-perovskite tandem solar mini-module. (e) *J*–*V* characteristic of the best-performing flexible all-perovskite tandem solar mini-module. The inset shows its photovoltaic parameters and stabilized power output under a fixed bias voltage of 9.6 V.

Building on the positive contribution of d-HLH to the performance of the NBG subcell, d-HLH-containing flexible TSCs outperformed the reference tandem cells (Fig. 4b and Table S3). Our best-performing tandem cell achieved a reverse-scan PCE of 23.6%, with a *V*_OC_ of 2.02 V, a *J*_SC_ of 14.77 mA cm^−2^, and an FF of 79.1%. The EQE spectra of the best-performing tandem cell are shown in Fig. S24a. The integrated current densities of the two subcells closely matched and agreed well with the *J*_SC_ obtained from the *J*–*V* measurements. The tandem device also delivered a stabilized PCE of 22.6% (Fig. S24b).

The operational stability of flexible all-perovskite TSCs was further evaluated *via* MPP tracking under continuous one-sun-equivalent LED illumination at ∼55 °C (Fig. S25). Fig. 4c shows the stability curves of the most stable reference and d-HLH tandem cells. The reference tandem cell retained 80% of its initial PCE after 139 h (*T*_80_ = 139 h). By contrast, the d-HLH tandem cell exhibited much improved operational stability, with an almost doubled *T*_80_ lifetime of 273 h. This improved operational stability aligns well with the better operational stability achieved in d-HLH-containing single-junction mixed Sn–Pb PSCs.

To demonstrate scalability, we further fabricated flexible all-perovskite tandem solar mini-modules using the developed laser scribing interconnection layout (Fig. 3d). An additional SnO_*x*_ layer was deposited by atomic layer deposition to serve as a conformal diffusion barrier (CDB) for better device stability.^46^ The overall mini-module configuration is shown in Fig. 4d. Reducing the *W*_d_ to 243 µm yielded a GFF of 96% (Fig. S26). The best-performing flexible all-perovskite tandem solar mini-module delivered a PCE of 20.6% over an aperture area of 10.8 cm^2^ (Fig. 4e). It also gave a stabilized PCE of 19.8% after 100 s of tracking at a fixed bias voltage of 9.6 V. The device delivered an average *V*_OC_ of 2.00 V, together with a *J*_SC_ of 13.74 mA cm^−2^ and an FF of 74.9%, per the series-connected tandem cell. The cell-normalized *V*_OC_ and *J*_SC_ were comparable to those of small-area tandem cells. The cell-to-module efficiency gap therefore arose primarily from the reduction in FF.

The operational stability of encapsulated flexible all-perovskite tandem solar mini-modules was also evaluated. In the two mini-modules tested, the more stable device exhibited a *T*_80_ lifetime of 76 h (Fig. S27). The observed cell-to-module loss in operational stability may arise from increased spatial nonuniformity upon upscaling and additional degradation pathways associated with laser-scribing-induced defects near the interconnection dead zones.^47,48^ The mechanical durability of flexible all-perovskite tandem solar mini-modules under different bending scenarios was investigated in our previous study.^12^

## Conclusions

In summary, we identified pronounced vertical morphological inhomogeneity in blade-coated mixed Sn–Pb perovskite films, associated with the through-thickness drying gradient established during gas quenching. This gradient generated a more defective upper region with smaller grains, which intensified nonradiative carrier recombination and constrained the performance of flexible mixed Sn–Pb PSCs. Incorporating d-HLH into the precursor ink regulated crystallization, enlarged the grains, and suppressed nonradiative carrier recombination. Consequently, the optimized flexible mixed Sn–Pb PSCs achieved a PCE of 18.4% with improved operational stability. Integrating the optimized mixed Sn–Pb subcell with a blade-coated WBG perovskite top subcell yielded flexible all-perovskite TSCs with a PCE of 23.6% and a *T*_80_ lifetime of 273 h under MPP tracking at ∼55 °C. Moreover, the flexible all-perovskite tandem solar mini-module achieved a stabilized PCE of 19.8%, demonstrating the scalability of the approach. Overall, this study identifies a critical quality-limiting factor in mixed Sn–Pb perovskite films prepared by gas-quenching-assisted doctor-blade coating and provides an additive-based strategy to mitigate it. These findings offer guidance for the scalable fabrication of high-performance flexible all-perovskite tandems.

## Experimental

### Materials

All materials were used as received without further purification. *N*,*N*-Dimethylformamide (DMF, 99.8%), dimethyl sulfoxide (DMSO, 99.5%), chlorobenzene (CB, 99.8%), isopropanol (IPA, 99.5%), propane-1,3-diammonium iodide (PDAI_2_, 98%), ethylenediammonium diiodide (EDAI_2_, 98%), tin iodide (SnI_2_, 99.99%), tin fluoride (SnF_2_, 99%), tin powder (99.5%), CsI (99.999%), lead thiocyanate (Pb(SCN)_2_), and Triton™ X-100 (10% in H_2_O) were purchased from Sigma-Aldrich Pty Ltd. PbI_2_ (99.5%), PbBr_2_ (99.999%) and (4-(3,6-dimethyl-9*H*-carbazol-9-yl)butyl)phosphonic acid (Me-4PACz, 99%) were purchased from Tokyo Chemical Industry Co., Ltd. Methylammonium iodide (MAI, >99.99%) and formamidinium iodide (FAI, >99.99%) were purchased from Greatcell Solar Materials (Australia). Fullerene (C_60_) and bathocuproine (BCP) were purchased from Xi'an Yuri Solar Co., Ltd (China). PEDOT:PSS (Clevios™ P VP AI 4083) was purchased from Heraeus Epurio LLC. Anhydrous ethanol (≥99.9%) was purchased from VWR International LLC. PEN/ITO substrates and NiO_*x*_ nanoparticles were purchased from Liaoning Youxuan New Energy Technology Co., Ltd (China). d-Homoserine lactone hydrochloride (d-HLH, 98%) was purchased from Apollo Scientific.

### Device fabrication

#### Solution preparation

The NBG Sn–Pb FA_0.7_MA_0.3_Pb_0.5_Sn_0.5_I_3_ perovskite precursor (1.8 M) was prepared by dissolving 414.9 mg PbI_2_, 335.3 mg SnI_2_, 216.7 mg FAI, 85.8 mg MAI, 7.1 mg SnF_2_ and 3 mg tin powder in 1 mL mixed solvent of DMF and DMSO (22 : 3, v/v). d-HLH (1 mol%) was added to the precursor solution. The perovskite precursor was stirred at 45 °C for 2 h and filtered through a 0.22 µm PTFE filter before use. The EDAI_2_ solution was prepared by dissolving 1 mg EDAI_2_ in 1 mL IPA and 1 mL CB.

The WBG FA_0.8_Cs_0.2_Pb(I_0.6_Br_0.4_)_3_ perovskite precursor (1.2 M) was prepared by dissolving 264.24 mg PbBr_2_, 221.28 mg PbI_2_, 165.12 mg FAI, 62.35 mg CsI, and 1.94 mg Pb(SCN)_2_ in 1 mL mixed solvent of DMF and DMSO (19 : 1, v/v). The perovskite precursor was stirred at 45 °C for 2 h and filtered through a 0.22 µm PTFE filter before use. The NiO_*x*_ dispersion was prepared by dissolving 12 mg NiO_*x*_ nanoparticles in 1 mL deionized water. The Me-4PACz solution was prepared by dissolving 1.2 mg Me-4PACz in 3 mL ethanol. The PDAI_2_ solution was prepared by dissolving 1 mg PDAI_2_ in 1 mL IPA.

The PEDOT:PSS solution was prepared by adding 60 µL Triton™ X-100 to 1 mL pristine PEDOT:PSS and then diluting the mixture with 2 mL IPA. The PEDOT:PSS solution was filtered through a 0.45 µm PVDF filter before use.

#### Fabrication of NBG PSCs

Prepatterned PEN/ITO substrates were fixed to rigid glass plates with adhesive. The substrates were sequentially sonicated for 15 min each in IPA, detergent solution, pure water, and IPA. They were dried under N_2_ flow and treated with UV-ozone (Jelight Company Inc.) for 30 min. PEDOT:PSS was spin-coated at 4000 rpm for 30 s with an acceleration of 1000 rpm s^−1^, followed by annealing at 130 °C for 20 min. Then, PEN/ITO/PEDOT:PSS stacks were immediately transferred to the glovebox for perovskite deposition. NBG perovskite ink (125 µL) was dispensed between the blade coater and the substrate. Wet films were blade-coated at 1 mm s^−1^ using a 250 µm coating gap and a quenching N_2_ stream under an optimal pressure of 2.0 bar. The films were immediately annealed on a preheated hotplate at 100 °C for 10 min. After cooling, EDAI_2_ was deposited onto the perovskite by blade coating at 5 mm s^−1^. Then, the perovskite films were transferred into a vacuum chamber for thermal evaporation. The films were transferred to a vacuum chamber for thermal evaporation. C_60_ (23 nm) and BCP (7 nm) were sequentially deposited below 3 × 10^−6^ mbar. Cu (100 nm) was then evaporated through a shadow mask below 3 × 10^−6^ mbar to form the top electrode. For characterization, the devices were encapsulated by edge-sealing a cover glass with a UV-curable encapsulant (Eversolar AB-341, Everlight Chemical).

#### Fabrication of flexible all-perovskite TSCs

Prepatterned PEN/ITO substrates were fixed to rigid glass plates with adhesive. The substrates were sequentially sonicated for 15 min each in IPA, detergent solution, pure water, and IPA. They were dried under N_2_ flow and treated with UV-ozone (Jelight Company Inc.) for 30 min. An aqueous NiO_*x*_ dispersion (12 mg mL^−1^) was spin-coated at 3000 rpm for 30 s, followed by annealing at 110 °C for 15 min. Then, PEN/ITO/NiO_*x*_ stacks were immediately transferred to the glovebox for SAM deposition. Me-4PACz solution (80 µL) was blade-coated onto PEN/ITO/NiO_*x*_ at 6 mm s^−1^ using a 250 µm coating gap, followed by gas quenching under a N_2_ stream at a pressure of 0.2 bar. The samples were annealed on a preheated hotplate at 100 °C for 10 min. After cooling, WBG perovskite ink (65 µL) was blade-coated at 6 mm s^−1^ using a 350 µm coating gap, followed by gas quenching under a N_2_ stream at a pressure of 1.2 bar. The films were immediately annealed on a preheated hotplate at 100 °C for 10 min. PDAI_2_ was deposited onto the perovskite by blade coating at 5 mm s^−1^. Then, the perovskite films were transferred into a vacuum chamber for thermal evaporation. The films were transferred to a vacuum chamber, where C_60_ (23 nm) was thermally evaporated below 3 × 10^−6^ mbar. The samples were then transferred to an atomic layer deposition (ALD) chamber. SnO_*x*_ was deposited at 100 °C using alternating pulses of tetrakis(dimethylamino)tin(iv) and deionized water for 200 cycles. Au clusters (1.2 nm) were deposited onto SnO_*x*_ by thermal evaporation at 0.02 Å s^−1^. The NBG perovskite bottom subcell was fabricated as described for the single-junction NBG PSCs up to the Cu deposition step. ALD-SnO_*x*_ was used to replace BCP for better device stability. For characterization, the devices were encapsulated by edge-sealing a cover glass with a UV-curable encapsulant (Eversolar AB-341, Everlight Chemical).

#### Fabrication of flexible all-perovskite tandem solar mini-modules

All deposition steps for module fabrication were identical to those used for cell fabrication unless otherwise specified. BCP was replaced with ALD-SnO_*x*_ to improve environmental resistance during P2 scribing. A 120 nm Cu top electrode was thermally evaporated without a shadow mask. Laser scribing was performed using a TruMark Station 5000 equipped with a 355 nm nanosecond-pulsed Nd:YVO_4_ laser. P1 scribing was performed on the PEN/ITO substrates before cleaning. P2 scribing was performed after depositing 150 ALD cycles of SnO_*x*_ on the bottom subcell. An additional 100-cycle SnO_*x*_ layer was then deposited as a conformal diffusion barrier. P3 scribing was performed after Cu deposition. Modules fabricated on 5 × 5 cm^2^ substrates comprised six cells, each 6 mm wide. P4 scribing defined a 10.8 cm^2^ aperture area for the solar mini-modules.

### Characterization studies

#### Current density–voltage (*J*–*V*) measurements

Cells and modules were measured under simulated AM1.5G illumination using a Keithley 2400 source meter in a four-wire configuration. A certified monocrystalline silicon reference cell (RS-ID-5, Fraunhofer ISE) was used to calibrate a class ABA solar simulator (LOT-Quantum Design) to one-sun AM1.5G illumination. Cells were measured at approximately 25 °C using an aperture mask that defined an active area of 0.057 cm^2^, whereas modules were measured without an aperture mask. Steady-state efficiency was determined by maximum-power-point tracking using a perturb-and-observe algorithm or current tracking at fixed bias.

#### External quantum efficiency (EQE)

The device external quantum efficiency (EQE) was measured using a lock-in amplifier. A chopped white-light probe (900 W halogen lamp, 280 Hz) was spectrally selected using a dual-grating monochromator. A certified monocrystalline silicon solar cell served as the reference. White bias illumination (∼0.1 sun) was applied during the calibration and measurement of single-junction cells. For tandem cells, 850 nm infrared and green LED bias lights were used to measure the EQE responses of the top and bottom subcells, respectively. The integrated *J*_SC_ was obtained by integrating the product of EQE and the AM1.5G photon flux.

#### Operational stability testing

Operational stability tests were performed using a Litos Lite platform (Fluxim, https://www.fluxim.com/litos-lite). Encapsulated devices were measured under continuous 1 sun-equivalent LED illumination and MPP tracking. The devices were measured in an environment with a relative humidity of approximately 10%. During continuous illumination, the device temperature gradually increased and stabilized at approximately 55 °C.

#### Scanning electron microscopy

Scanning electron microscopy (SEM) images were acquired using a Hitachi S-4800 microscope operated at an accelerating voltage of 5 kV. To expose buried interfaces, a UV-curable adhesive was applied to the perovskite surface, covered with a glass plate, and cured under ultraviolet illumination. After curing, the substrate was slowly peeled away, preferentially delaminating mechanically weak buried interfaces and exposing the underlying surfaces for SEM imaging. For grain-size analysis, grain boundaries were manually delineated, and enclosed regions were identified as individual grains using AI-assisted image analysis. Grain-size distributions were calculated from the equivalent circular diameters of all analyzed grains.

#### Steady-state and time-resolved photoluminescence

Steady-state photoluminescence (PL) and time-resolved photoluminescence (TRPL) were measured using a MicroTime 100 system (PicoQuant). The test samples consisted of a PEN/ITO/PEDOT:PSS/perovskite multilayer structure and were encapsulated using glass and edge-sealing to protect them from exposure to ambient air. A 639 nm pulsed laser diode with an approximately 100 ps pulse width was used for excitation. For steady-state PL measurements, glass-encapsulated perovskite films were excited through either the PEN substrate or the perovskite surface, as specified. Emission spectra were collected using a near-infrared detector from 800 to 1200 nm with a 1 nm step size. For TRPL measurements, the laser repetition rate was 0.5 MHz. Acquisition was stopped when the maximum photon counts in a single time channel reached 10 000. The TRPL decay curves were fitted using a biexponential decay function.

#### X-ray diffraction (XRD)

XRD patterns were collected using a PANalytical X'Pert PRO diffractometer equipped with a PW3050/60 *θ*/2*θ* goniometer and a PW3064 spinner sample stage. Measurements were performed using Cu Kα radiation over a 2*θ* range of 5° to 60° with a step size of 0.0167°.

#### Time-of-flight secondary ion mass spectrometry (ToF-SIMS)

ToF-SIMS depth profiles were acquired using a ToF-SIMS V instrument (IONTOF). A 25 keV Bi^3+^ primary ion beam was operated at 0.31 pA over a 50 × 50 µm^2^ raster. A 1000 eV Cs^+^ ion beam with a pulse current of 34 nA was rastered over 200 × 200 µm^2^ to sputter the film. The secondary-ion spectra were normalized to the total ion counts.

#### Other characterization methods

Dark *J*–*V*, capacitance–voltage (*C*–*V*), electrochemical impedance spectroscopy (EIS), and transient photovoltage (TPV) measurements were performed using a PAIOS characterization system. Dark *J*–*V* curves were recorded by sweeping the applied voltage from +1.0 to −0.5 V. EIS measurements were performed at the maximum-power-point voltage from 10 MHz to 10 Hz using a 6 mV AC amplitude. For TPV measurements, the steady-state bias-light intensity was set to 100%, and a 2% light pulse of 50 µs duration was applied. The subsequent acquisition time was 1 s.

## Author contributions

J. L.: conceptualization, data curation, formal analysis, investigation, methodology, validation, visualization, writing – original draft and writing – review & editing; J. S. A.: formal analysis, methodology, supervision and writing – review & editing; A. W.: investigation and writing – review & editing; C. S.: supervision and writing – review & editing; F. F.: conceptualization, funding acquisition, project administration, supervision and writing – review & editing.

## Conflicts of interest

There are no conflicts to declare.

## Supplementary Material

EL-OLF-D6EL00168H-s001

EL-OLF-D6EL00168H-s002

## Data Availability

The data supporting this article have been included as part of the supplementary information (SI). Supplementary information: Fig. S1–S27, Note S1, and Tables S1–S3. See DOI: https://doi.org/10.1039/d6el00168h.
