# Supplementary material for: Mitigating vertical crystallization inhomogeneity in blade-coated Sn–Pb perovskites for flexible all-perovskite tandem modules
Source: EES Solar. 2026 Oct 23. Online ahead of print. doi: 10.1039/d6el00168h (PMC13733839; doi:10.1039/d6el00168h)
Supplement: Supplementary file 1 [file EL-OLF-D6EL00168H-s002.pdf]

Supplementary Information (SI) for EES Solar.
This journal is © The Royal Society of Chemistry
Supplementary Information

Mitigating Vertical Crystallization Inhomogeneity in Blade-Coated Sn-Pb Perovskites for Flexible All-Perovskite Tandem Modules

Jincheng Luo[a], Jonathan S. Austin[a], Amber Wright[a], Chih-Jen Shih[b], Fan Fu[a,*]

[a]Laboratory for Thin Films and Photovoltaics, Empa - Swiss Federal Laboratories for Materials Science and Technology, Dübendorf, Switzerland

[b]Institute for Chemical and Bioengineering, ETH Zürich, Zürich, Switzerland

[*]Corresponding author: Fan Fu, fan.fu@empa.ch

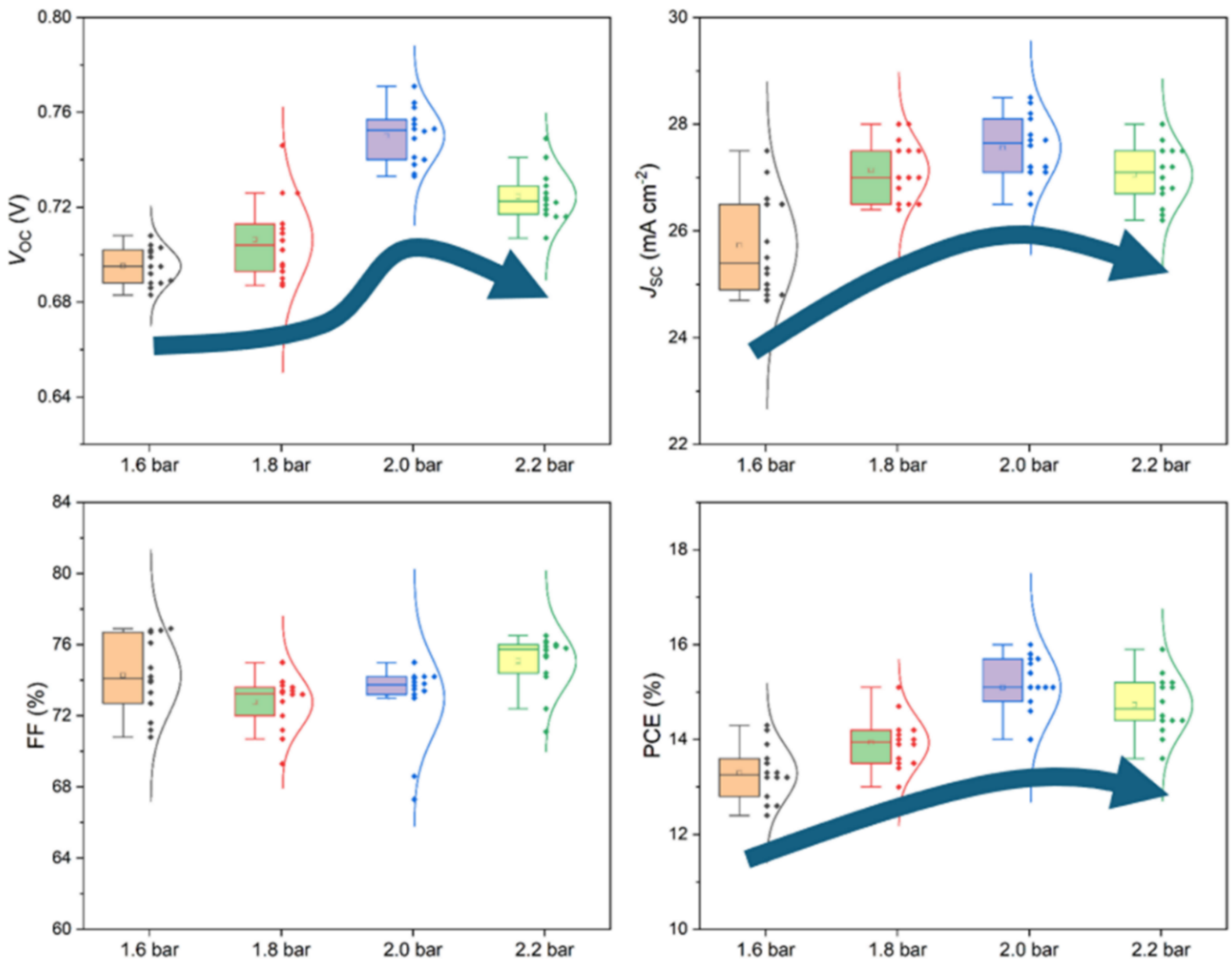

Fig. S1: Statistical PV parameters of flexible mixed Sn-Pb PSCs under different quenching pressure, containing 14 cells for each group from single batch.

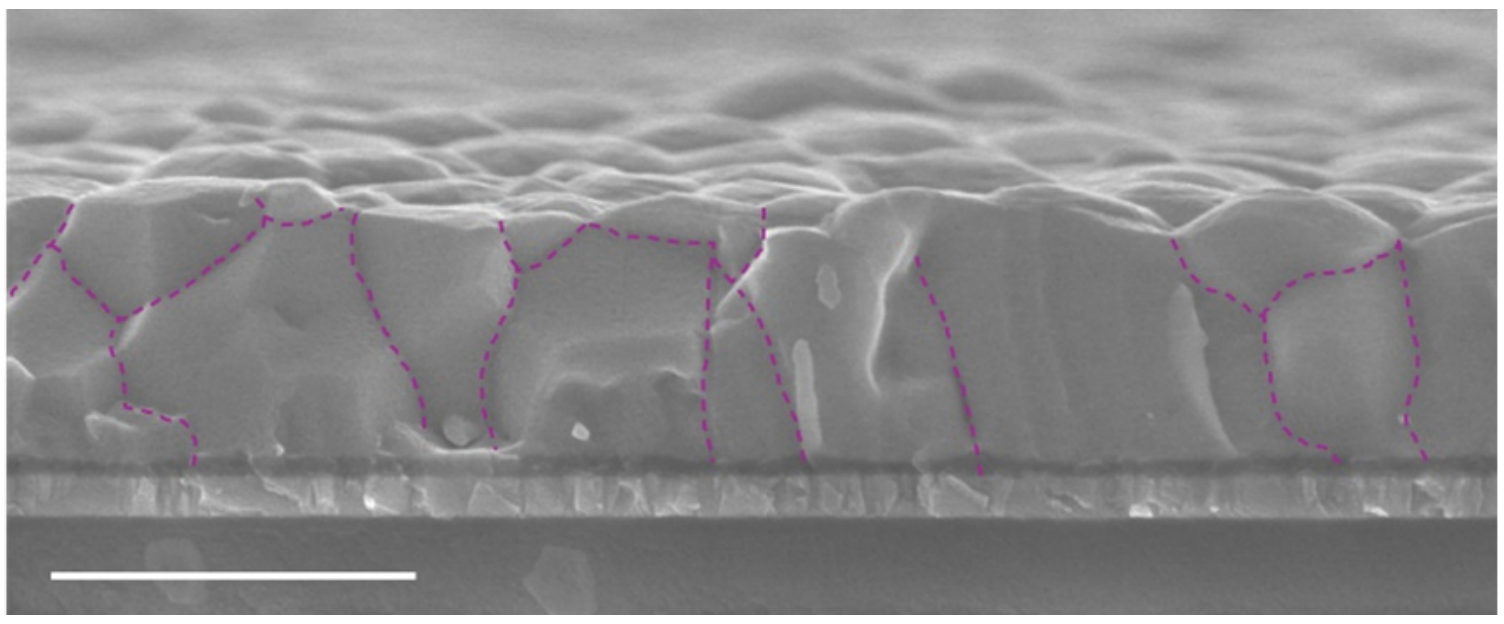

Fig. S2: Cross-sectional SEM image of gas-quenching-assisted blade-coated rigid mixed Sn-Pb perovskite film. Scale bar: 1 μm.

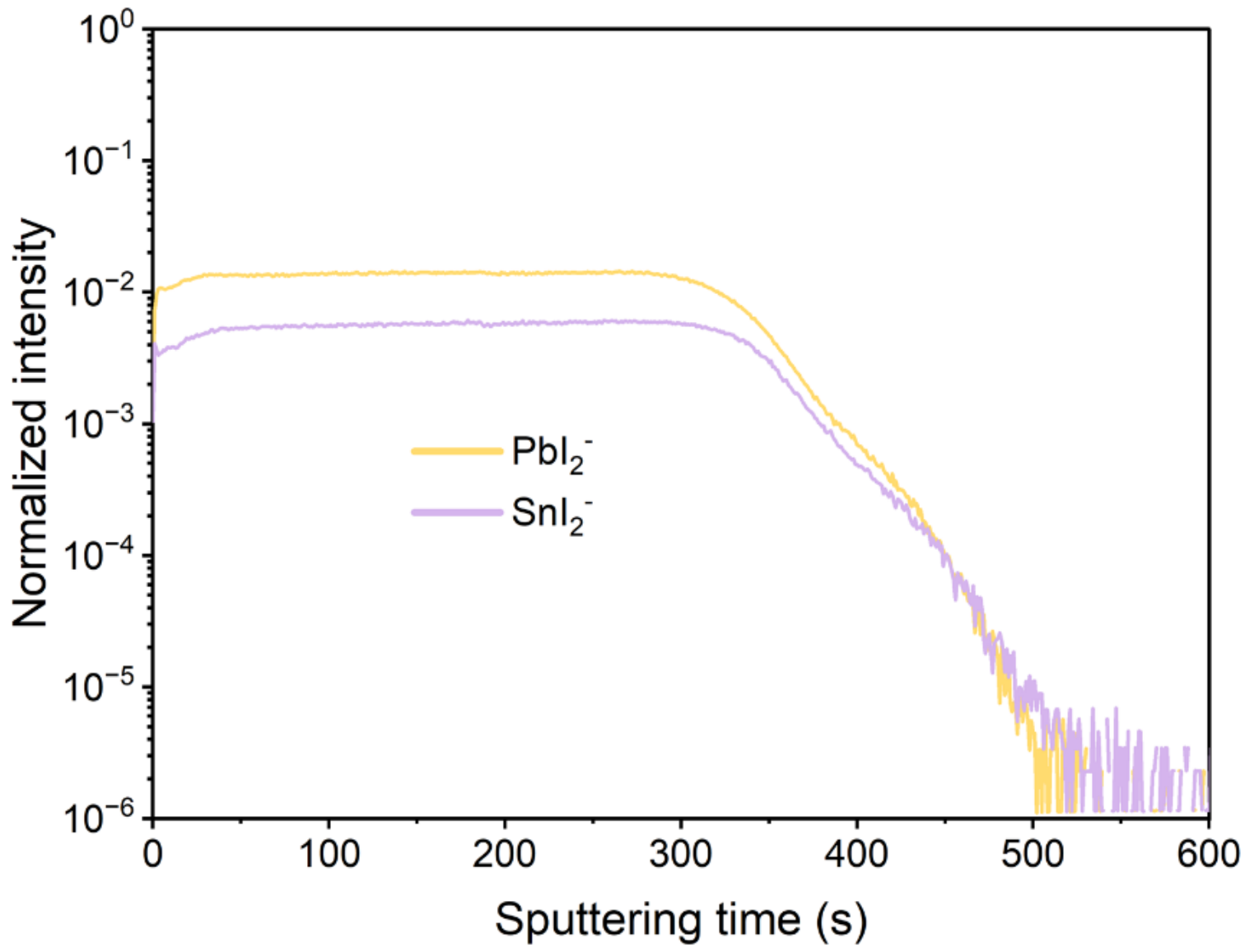

Fig. S3: SnI_2^-/PbI_2^- distribution in gas-quenching-assisted blade-coated mixed Sn-Pb perovskite films, acquired from ToF-SIMS study.

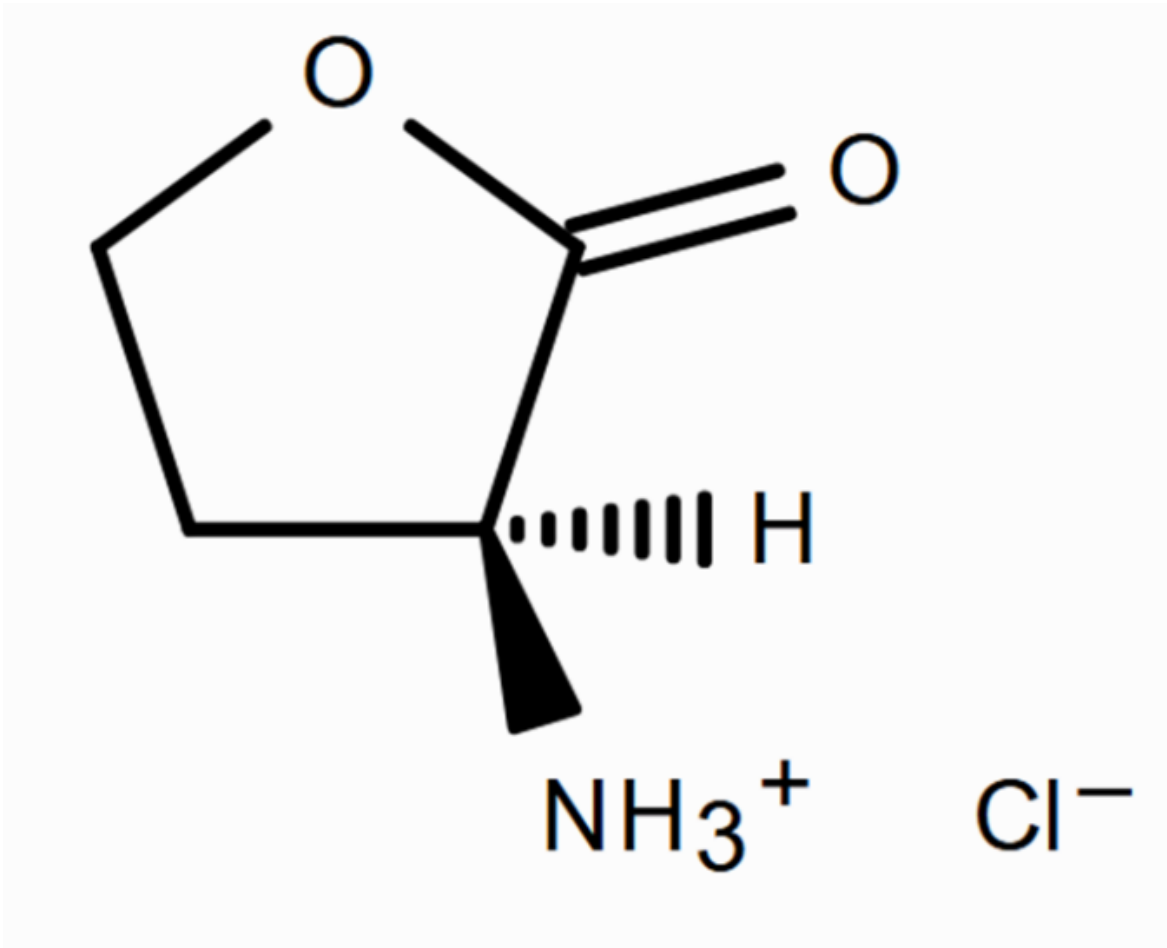

Fig. S4: Chemical structure of additive D-HLH.

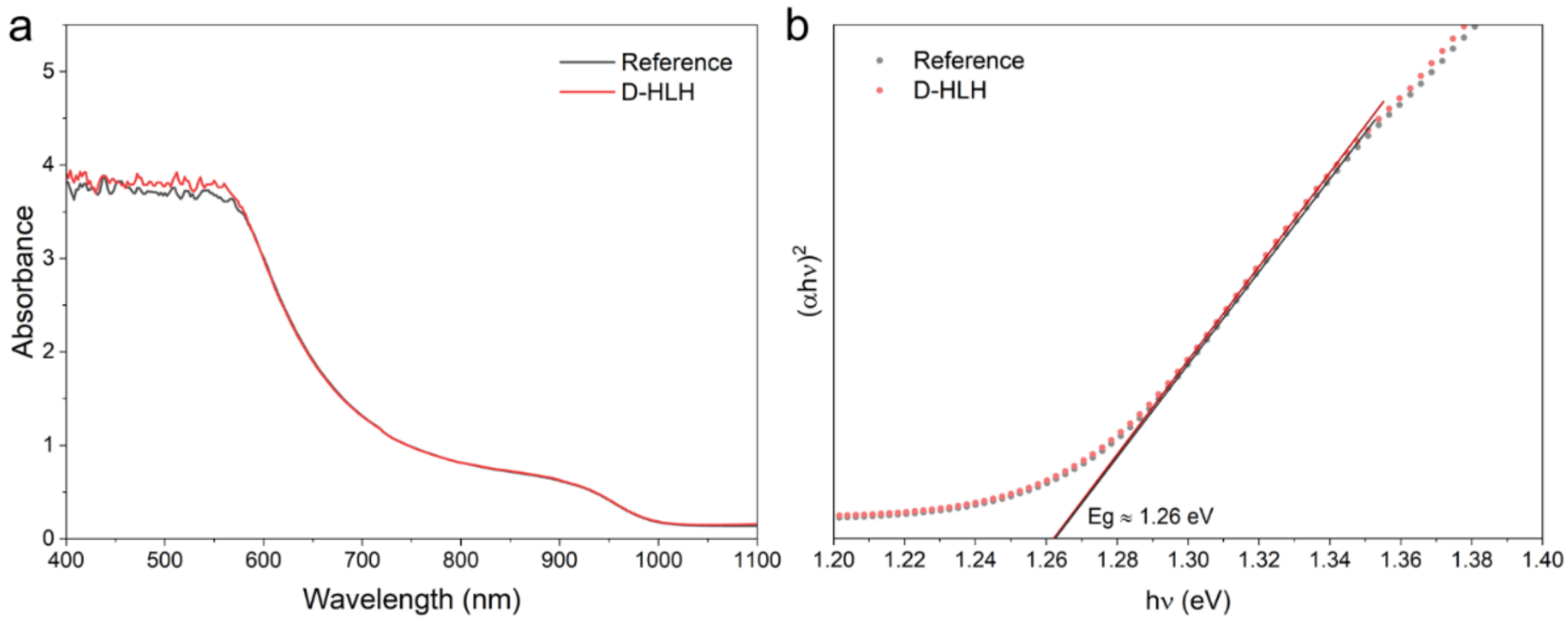

Fig. S5: **(a)** Absorbance spectra and **(b)** Tauc plots of the reference and D-HLH flexible mixed Sn-Pb perovskite films.

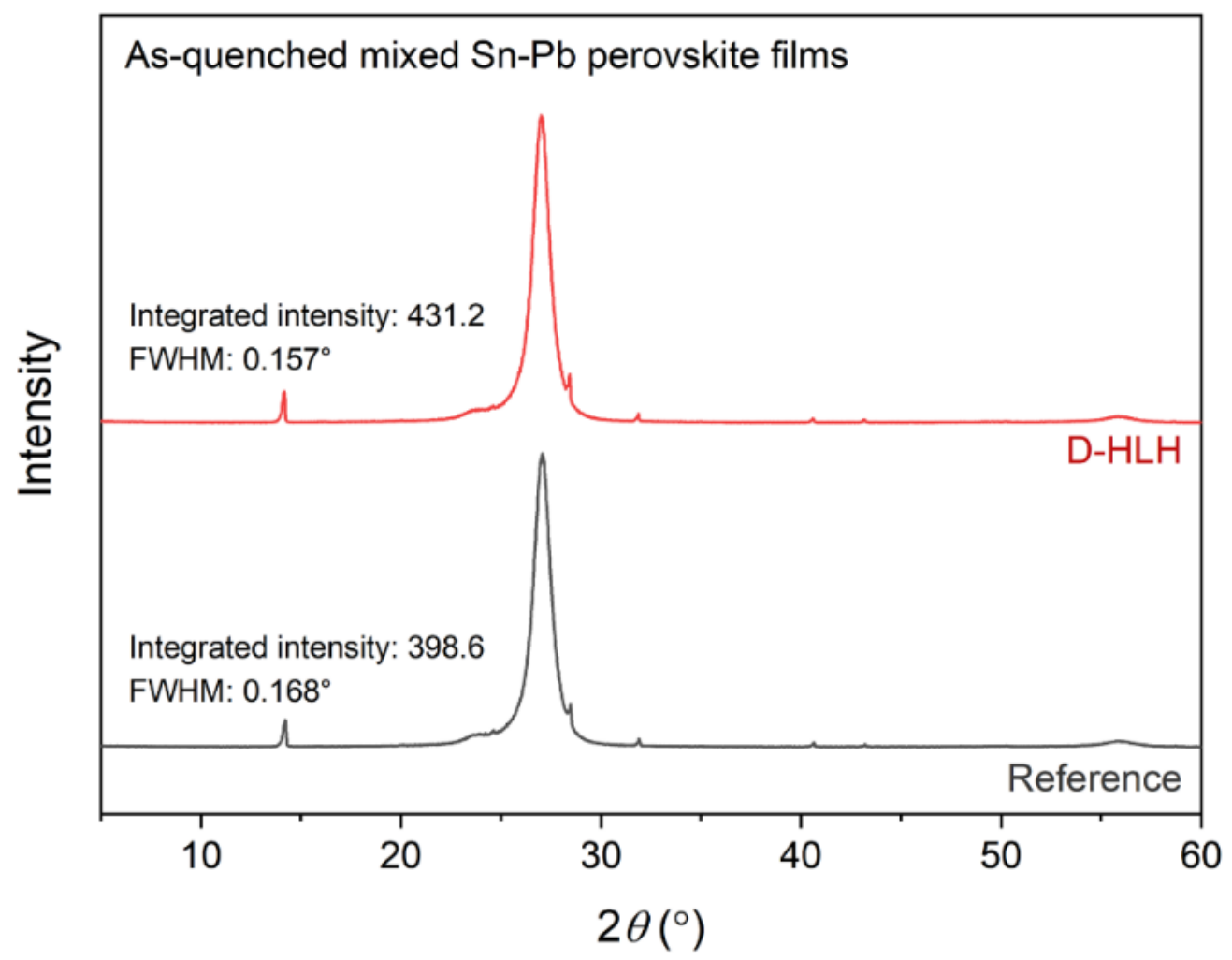

Fig. S6: XRD patterns of as-quenched flexible mixed Sn-Pb perovskite films.

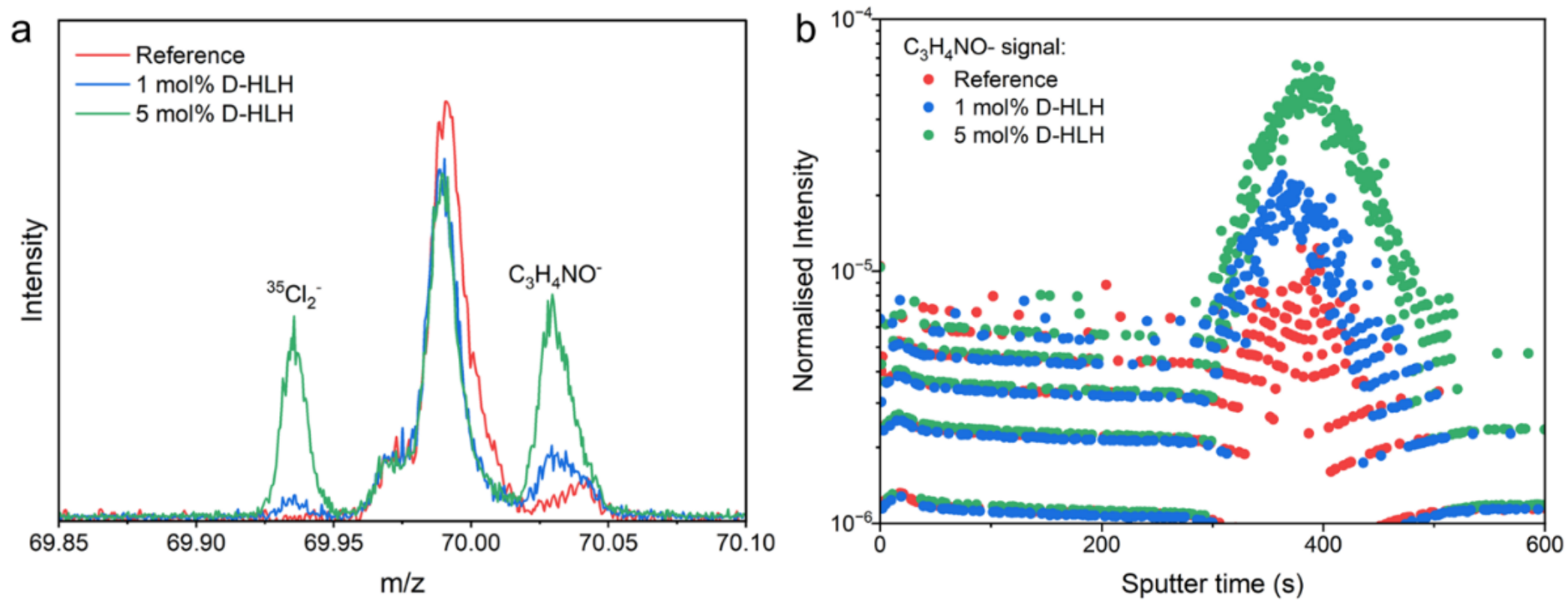

Fig. S7: **(a)** Mass spectrum of mixed Sn-Pb perovskite films at different D-HLH concentrations, acquired from ToF-SIMS study. **(b)** ToF-SIMS depth profile of $C_3H_4NO^-$ in mixed Sn-Pb perovskite films at different D-HLH concentrations.

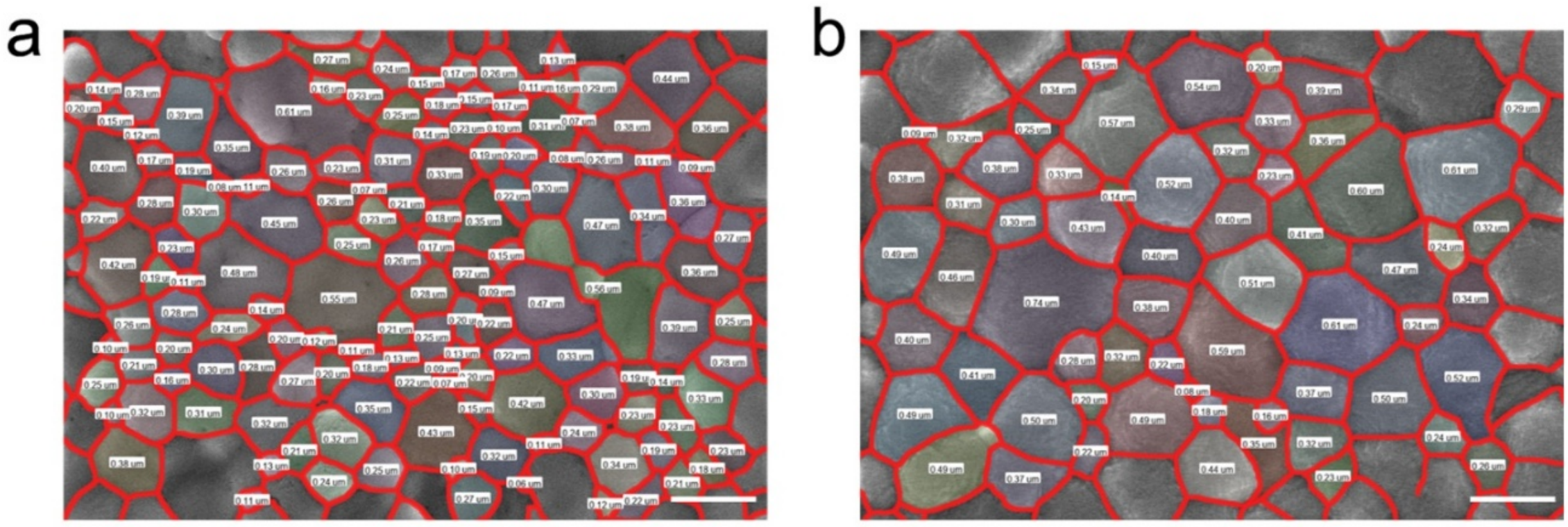

Fig. S8: Processed top-view SEM images of **(a)** reference and **(b)** D-HLH mixed Sn-Pb perovskite films. Red outlines delineate clearly resolved grain boundaries, and the labels indicate the equivalent circular diameter of each enclosed grain. Scale bar: 500 nm.

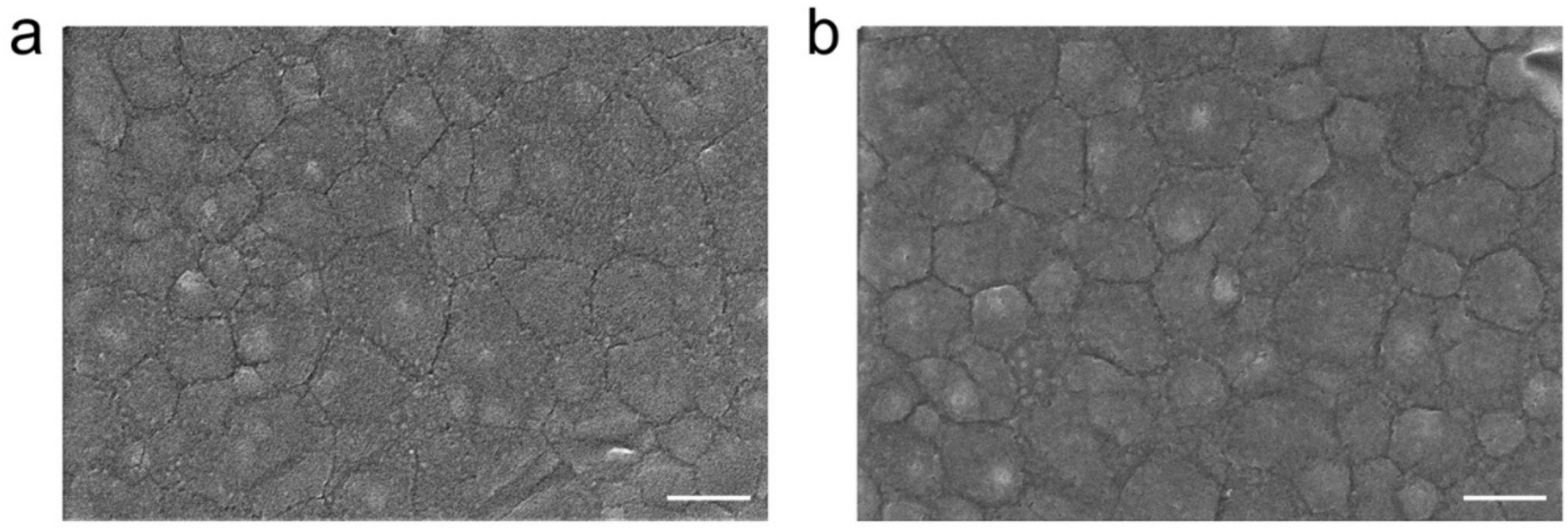

Fig. S9: Bottom-view SEM images of **(a)** reference and **(b)** D-HLH mixed Sn-Pb perovskite films. Scale bar: 500 nm.

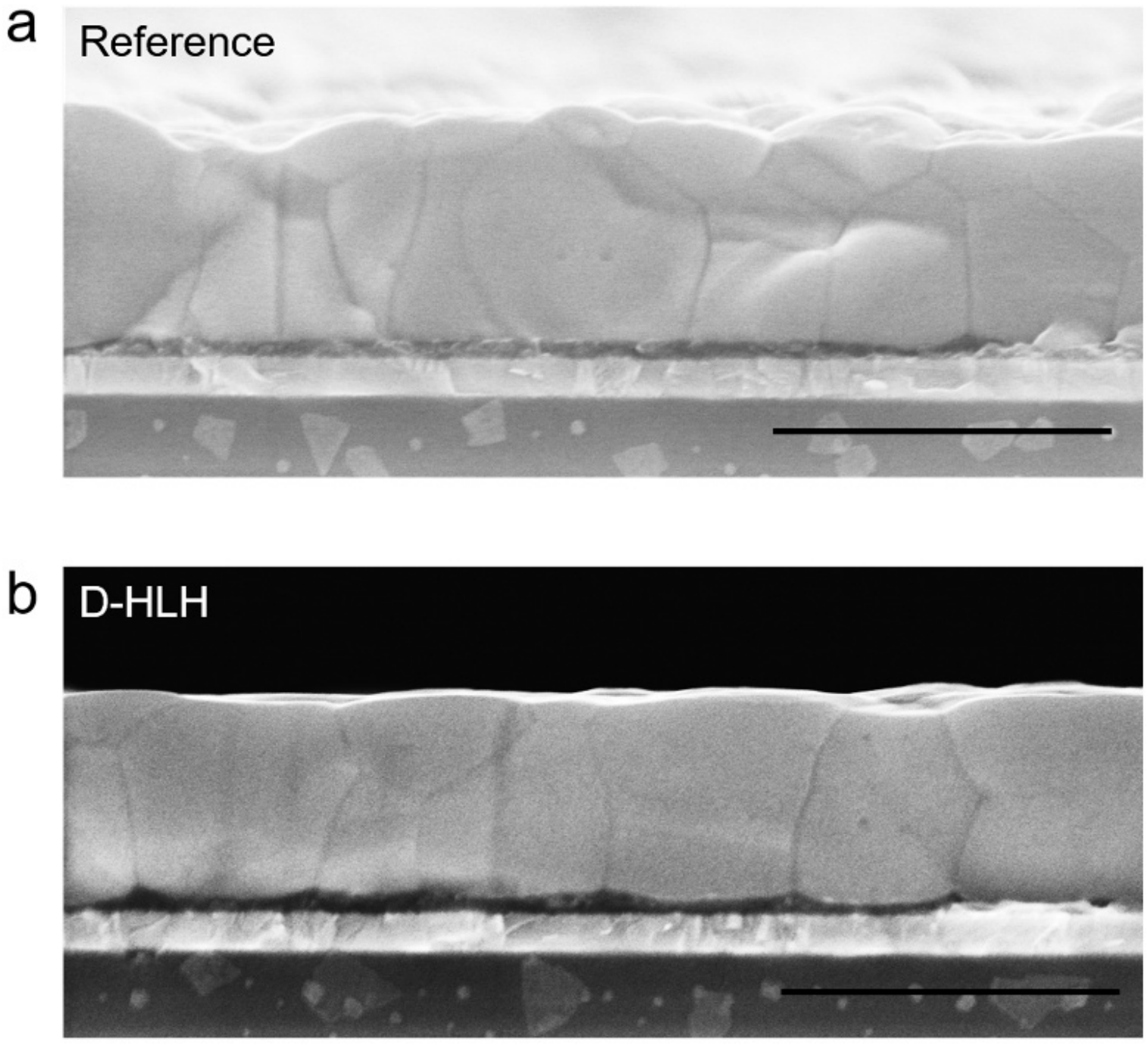

Fig. S10: Cross-sectional SEM images of **(a)** reference and **(b)** D-HLH mixed Sn-Pb perovskite films coated on rigid glass/ITO/PEDOT:PSS. Scale bar: 1 μm.

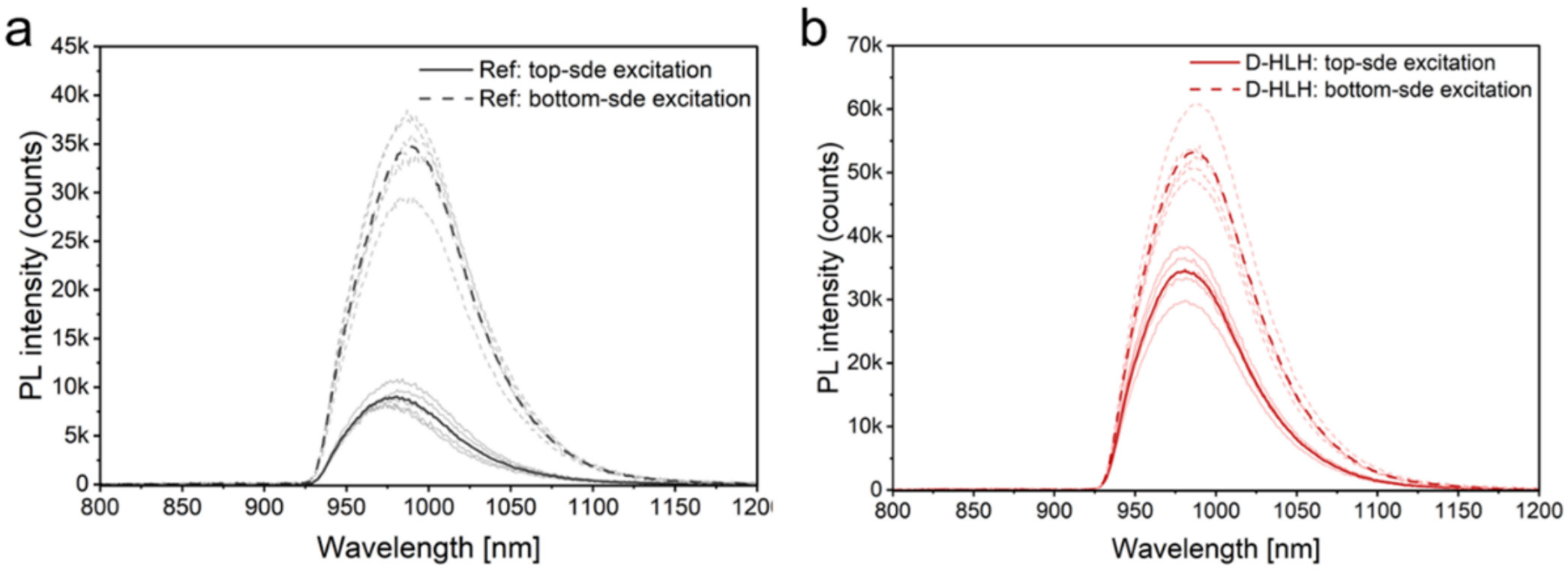

Fig. S11: PL spectra of **(a)** reference and **(b)** D-HLH mixed Sn-Pb perovskite films, measured from 5 spots across 5 × 5 cm^2 substrates, excited from either the perovskite or PEN side. The thin lines show the spectra measured at each spot, and the bold lines show the average spectra.

Note S1: Quantitative analysis of observed bottom/top PL intensity ratio.

The difference in PL intensity between top-side and bottom-side excitation can arise not only from the depth-dependent optoelectronic quality of the perovskite film but also from geometry-dependent optical effects, including excitation-side reflectance, parasitic absorption by the substrate/transport layers, and self-absorption of the emitted PL. We therefore evaluated the contributions of these optical effects to the observed bottom/top PL intensity ratio.

Firstly, we evaluated the difference in excitation-light reflection using the reflectance spectra of the flexible mixed Sn-Pb perovskite films measured from the PEN and perovskite sides (Fig. S12a). At the excitation wavelength of 639 nm, approximately 16.0% and 25.0% of the incident light is reflected for PEN-side and perovskite-side incidence, respectively. Secondly, Parasitic absorption by the PEN/ITO/PEDOT:PSS stack was evaluated from its measured reflectance and transmittance at 639 nm and was determined to be approximately 6.5% (Fig. S12b). Taking these experimentally determined optical losses into account, the expected bottom/top ratio in effective excitation intensity is approximately 1.03, estimated as (1-16.0%-6.5%)/(1-25.0%).

For reabsorption/self-absorption, we expect it to have negligible differences between the reference and D-HLH mixed Sn-Pb perovskite films since we see no significant differences between their absorption spectra (Fig. S5). Assuming that the absorption coefficient is consistent at different depths, the expected bottom/top ratio in PL intensity loss caused by self-absorption is 1.

Importantly, the experimentally observed bottom/top PL intensity ratio is substantially larger than the difference expected solely from the quantified optical losses and self-absorption. The measured PL intensity ratio is 3.85 ± 0.53 for reference film and decreases to 1.55 ± 0.17 after D-HLH addition. Moreover, the reference and D-HLH films were measured using the same substrate/HTL stack and identical excitation. Thus, such substantial reduction of bottom/top PL intensity ratio suggests improved vertical homogeneity arising from D-HLH-modulated crystallization.

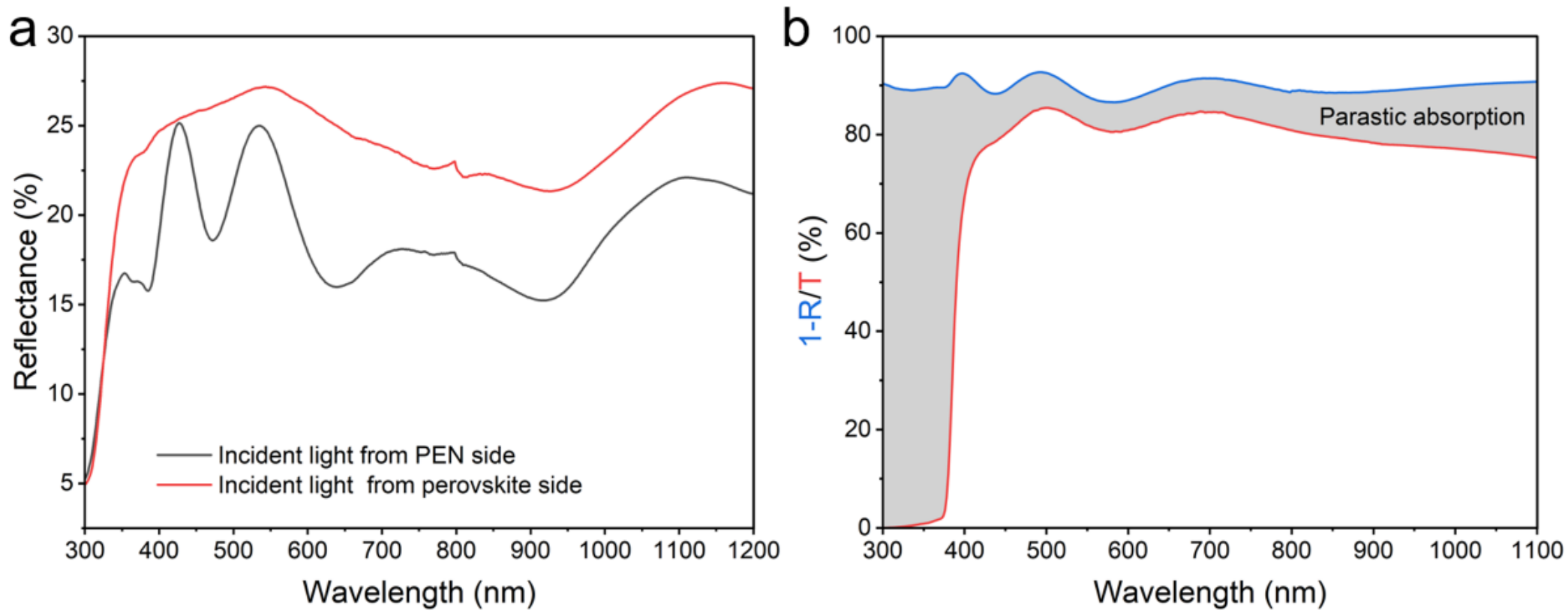

Fig. S12. **(a)** Reflectance spectra of encapsulated flexible mixed Sn-Pb perovskite films measured with light incident from the PEN side and the perovskite side. **(b)** Parasitic absorption spectrum of the PEN/ITO/PEDOT:PSS stack, calculated from the corresponding reflectance and transmittance spectra measured with light incident from the PEN side.

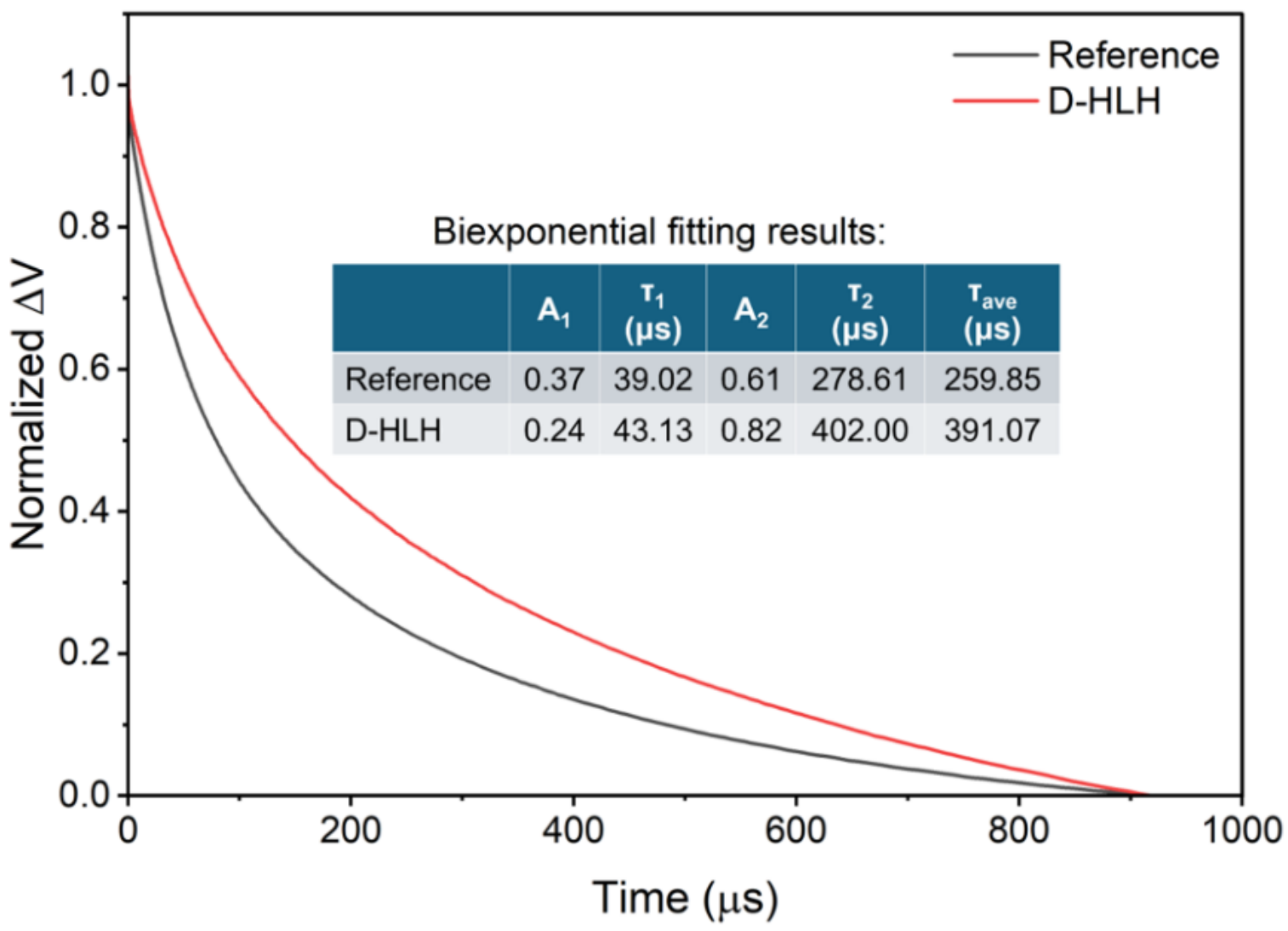

	A_1	τ_1 (μs)	A_2	τ_2 (μs)	τ_{ave} (μs)
Reference	0.37	39.02	0.61	278.61	259.85
D-HLH	0.24	43.13	0.82	402.00	391.07

Fig. S13: Transient photovoltage (TPV) decay curves of reference and D-HLH mixed Sn-Pb PSCs.

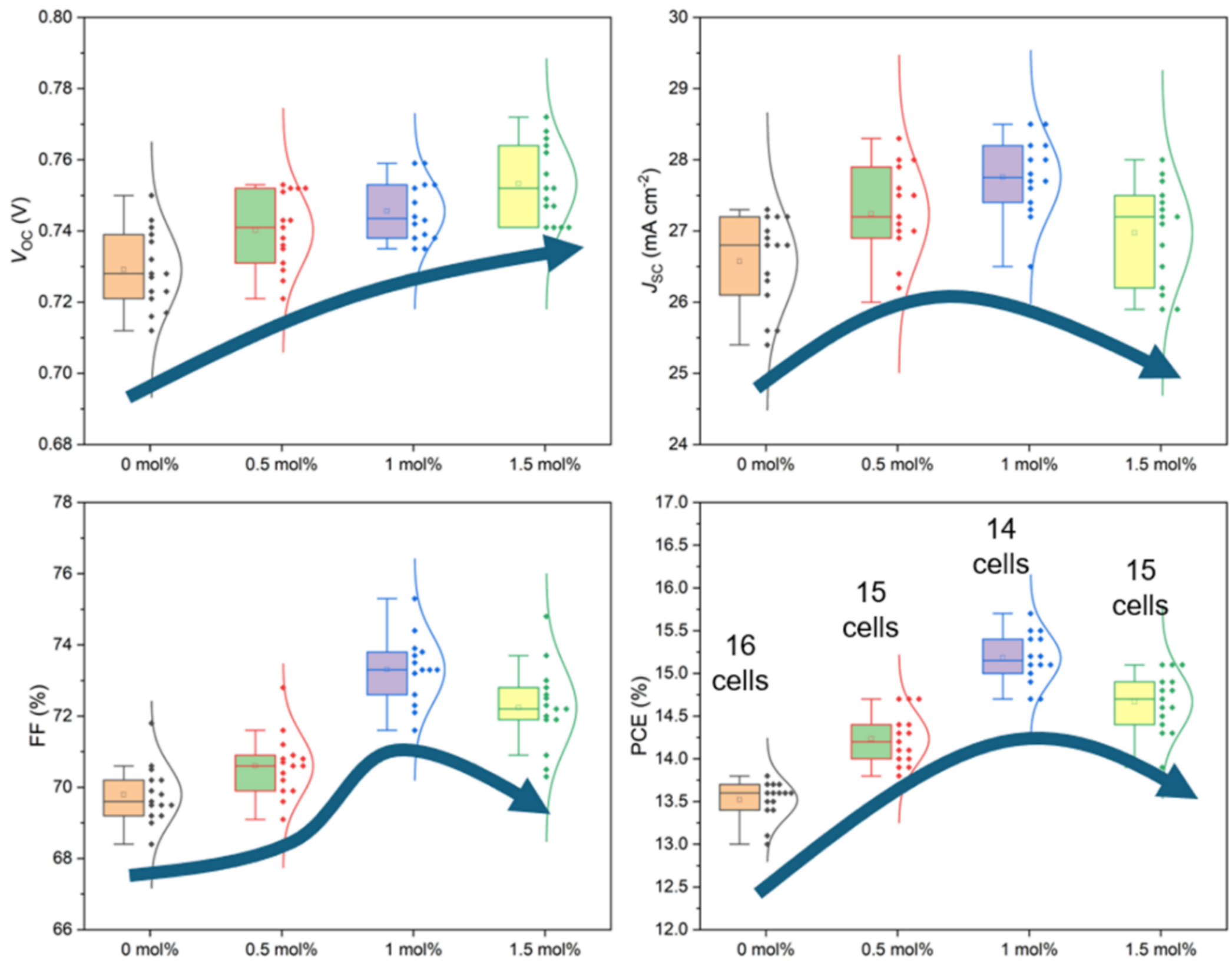

Fig. S14: Statistical PV parameters of mixed Sn-Pb PSCs at different D-HLH concentrations. These cells were fabricated from single batch.

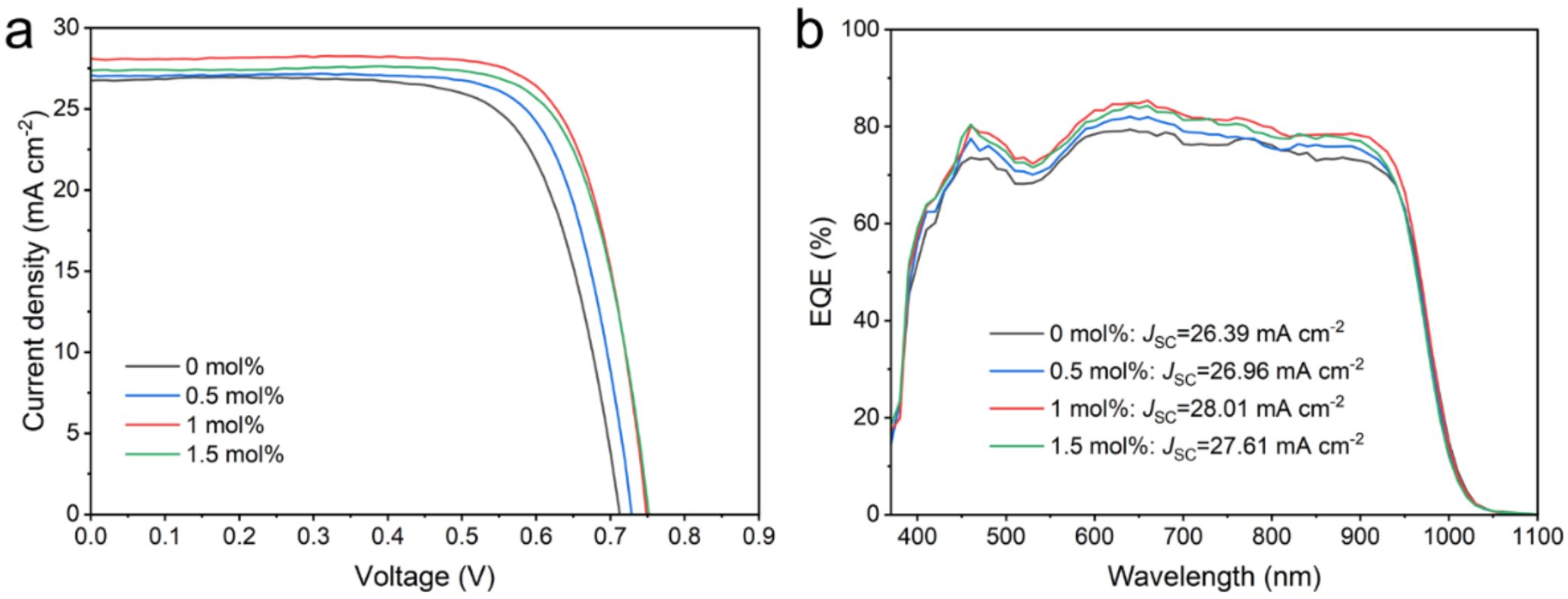

Fig. S15: **(a)** *J-V* curves from reverse scan and **(b)** EQE spectra of representative flexible mixed Sn-Pb PSCs at different D-HLH concentration.

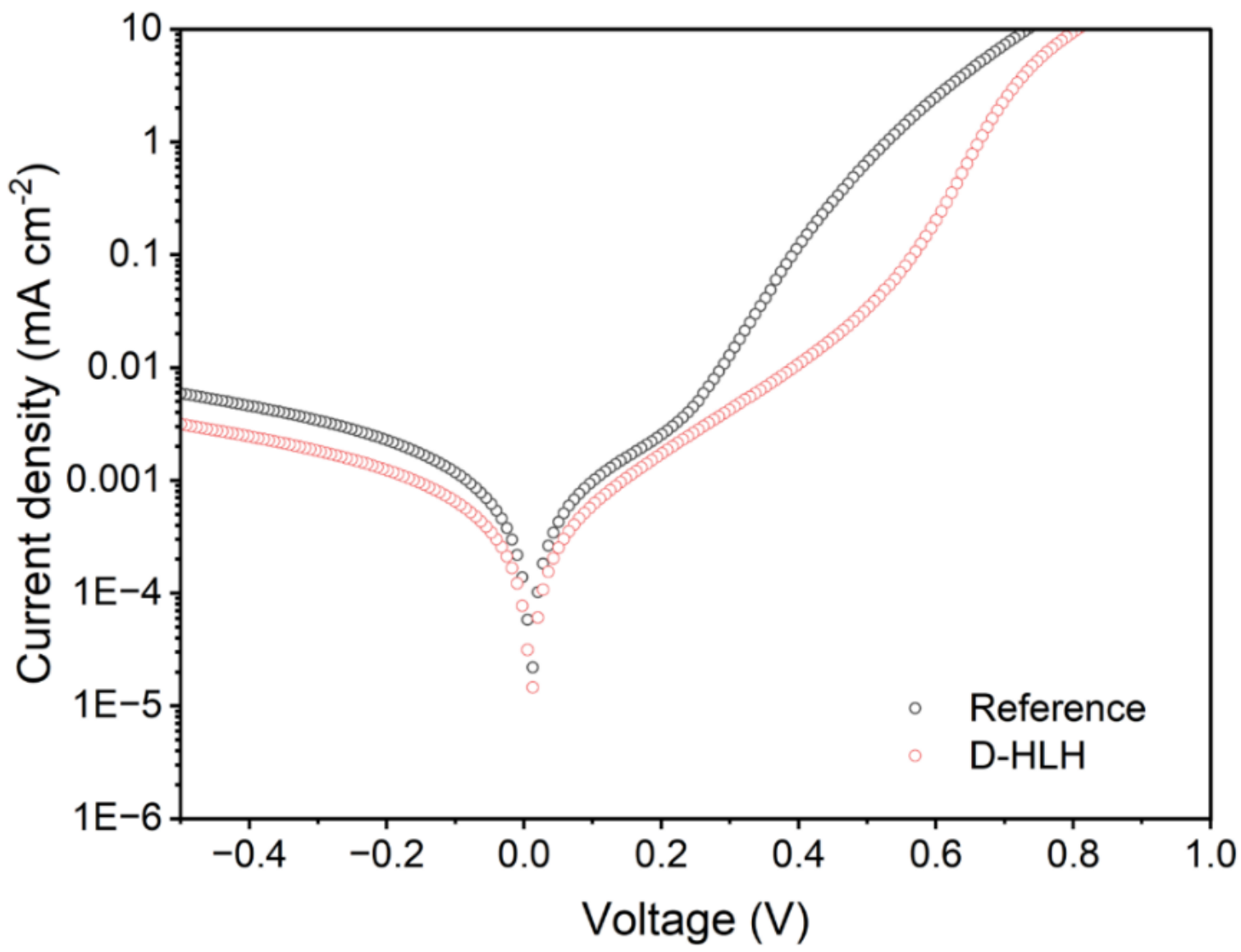

Fig. S16: Dark *J-V* curves of reference and D-HLH mixed Sn-Pb PSCs.

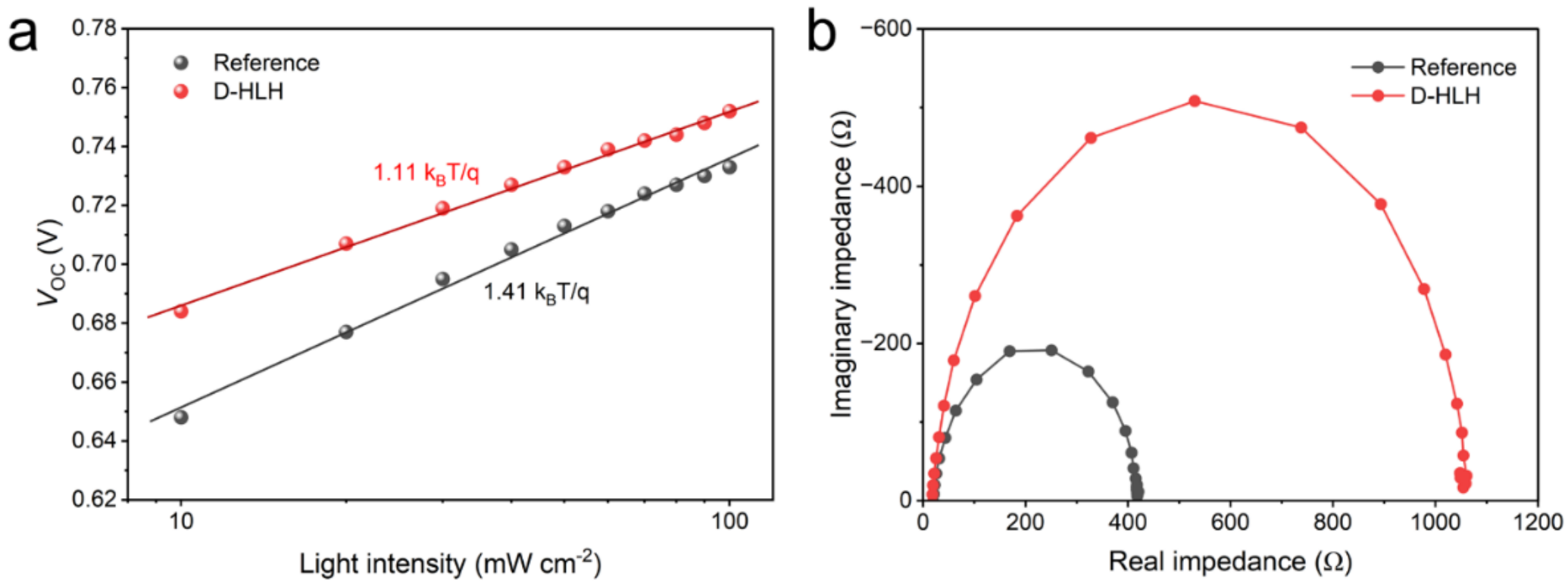

Fig. S17: **(a)** Light intensity dependent V_{OC} of reference and D-HLH mixed Sn-Pb PSCs. **(b)** Nyquist plots of reference and D-HLH mixed Sn-Pb PSCs, obtained from electrochemical impedance spectroscopy measurements.

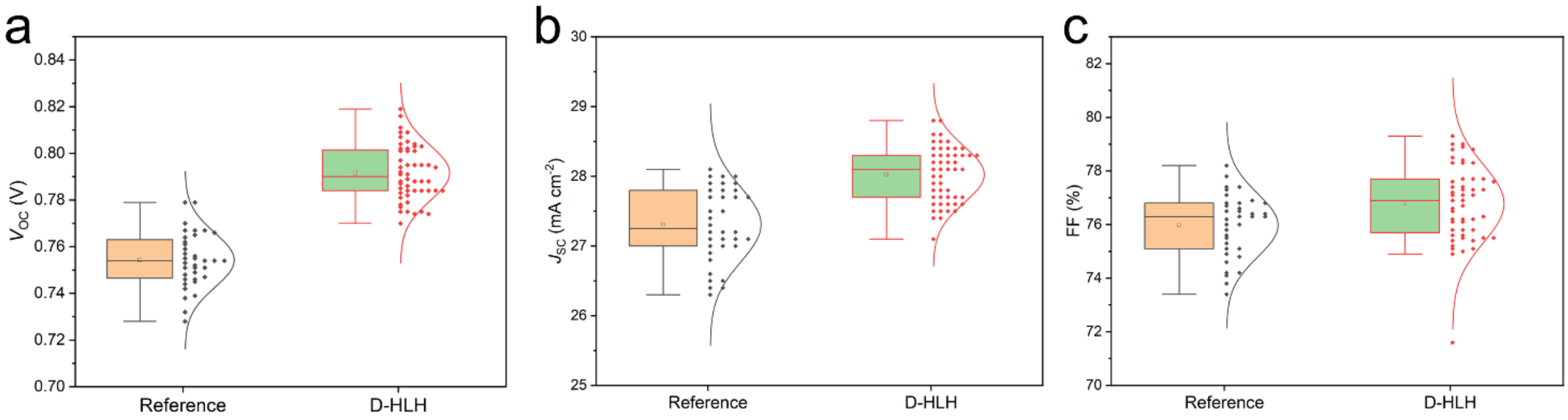

Fig. S18: Statistical **(a)** V_{OC}, **(b)** J_{SC}, and **(c)** FF of flexible mixed Sn-Pb PSCs after $EDAI_2$ PDT, including 36 reference and 52 D-HLH cells from multiple batches.

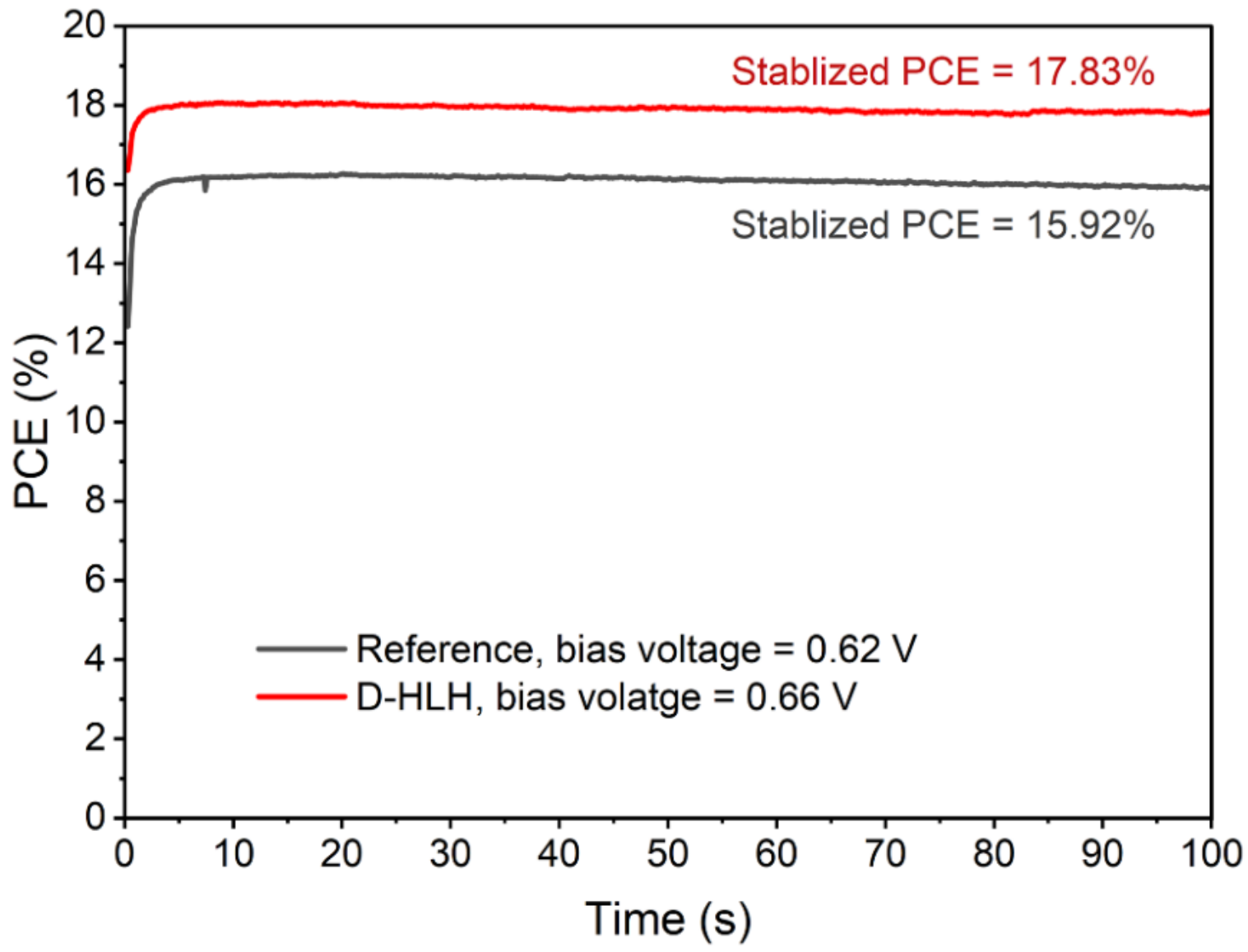

Fig. S19: Stabilized power output of flexible mixed Sn-Pb PSCs at fixed bias voltage.

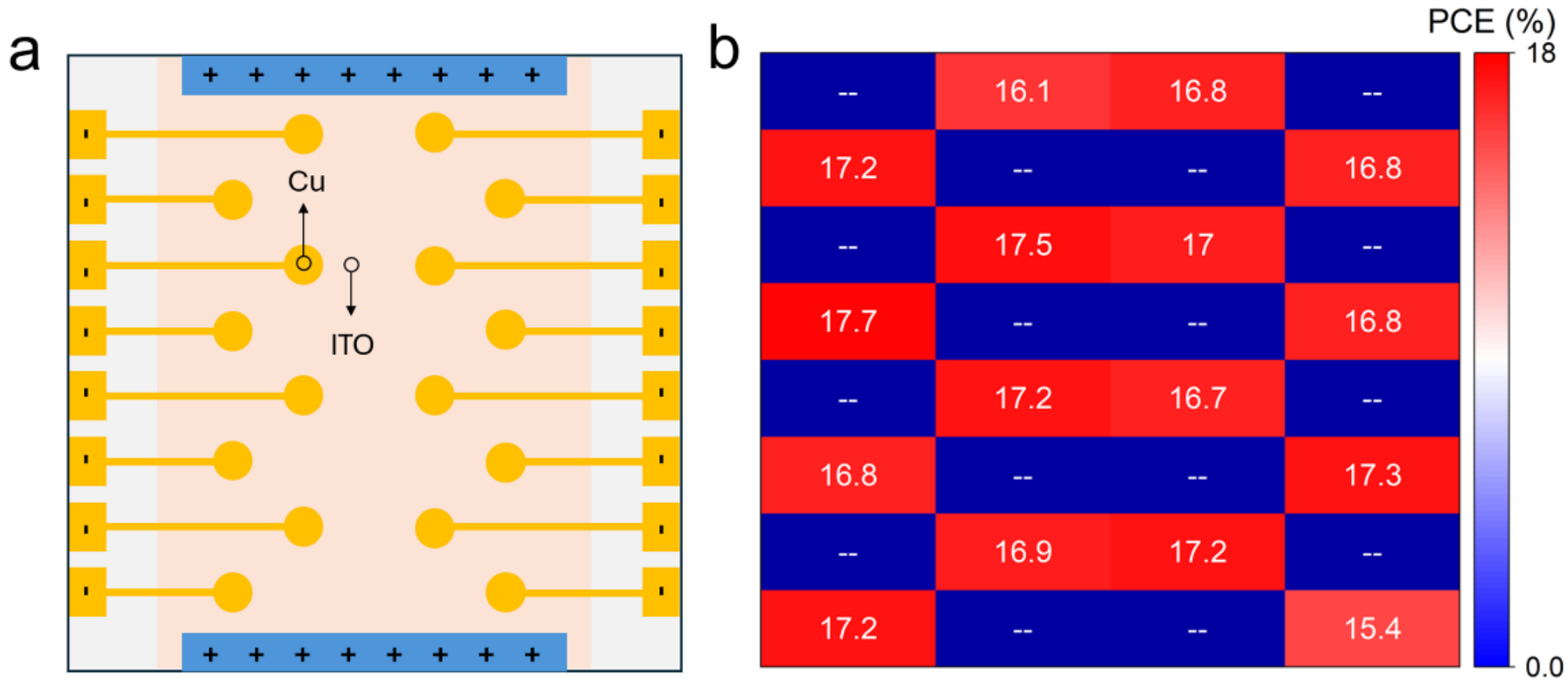

Fig. S20: **(a)** Schematic pattern of flexible WBG PSCs fabricated on 5 × 5 cm² flexible PEN/ITO substrates, containing 16 pixels. **(b)** PCE heatmap of representative D-HLH flexible mixed Sn-Pb PSCs fabricated on single 5 × 5 cm² PEN/ITO substrate.

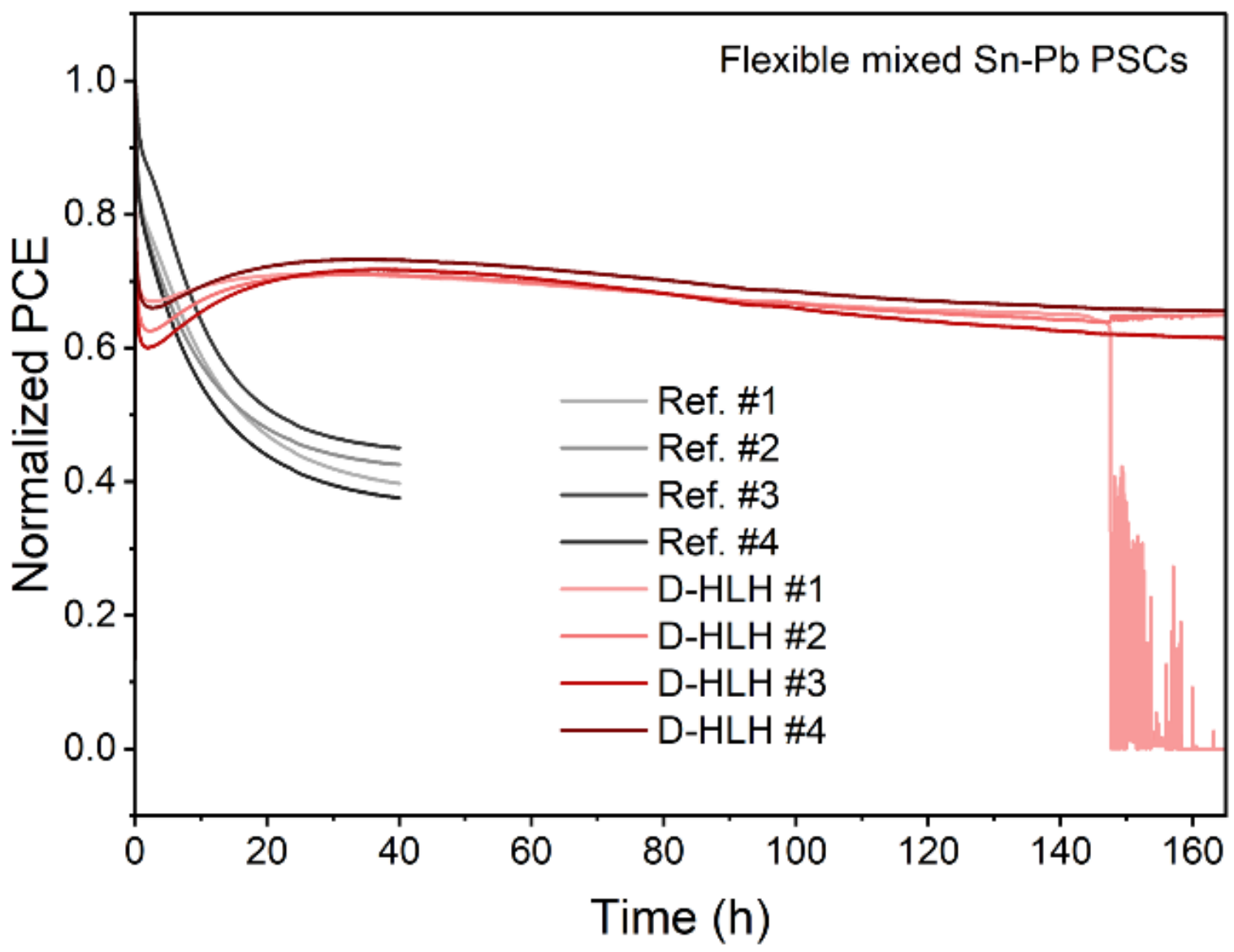

Fig. S21: Operational stability of reference and D-HLH flexible mixed Sn-Pb PSCs under MPP tracking at ~55 °C.

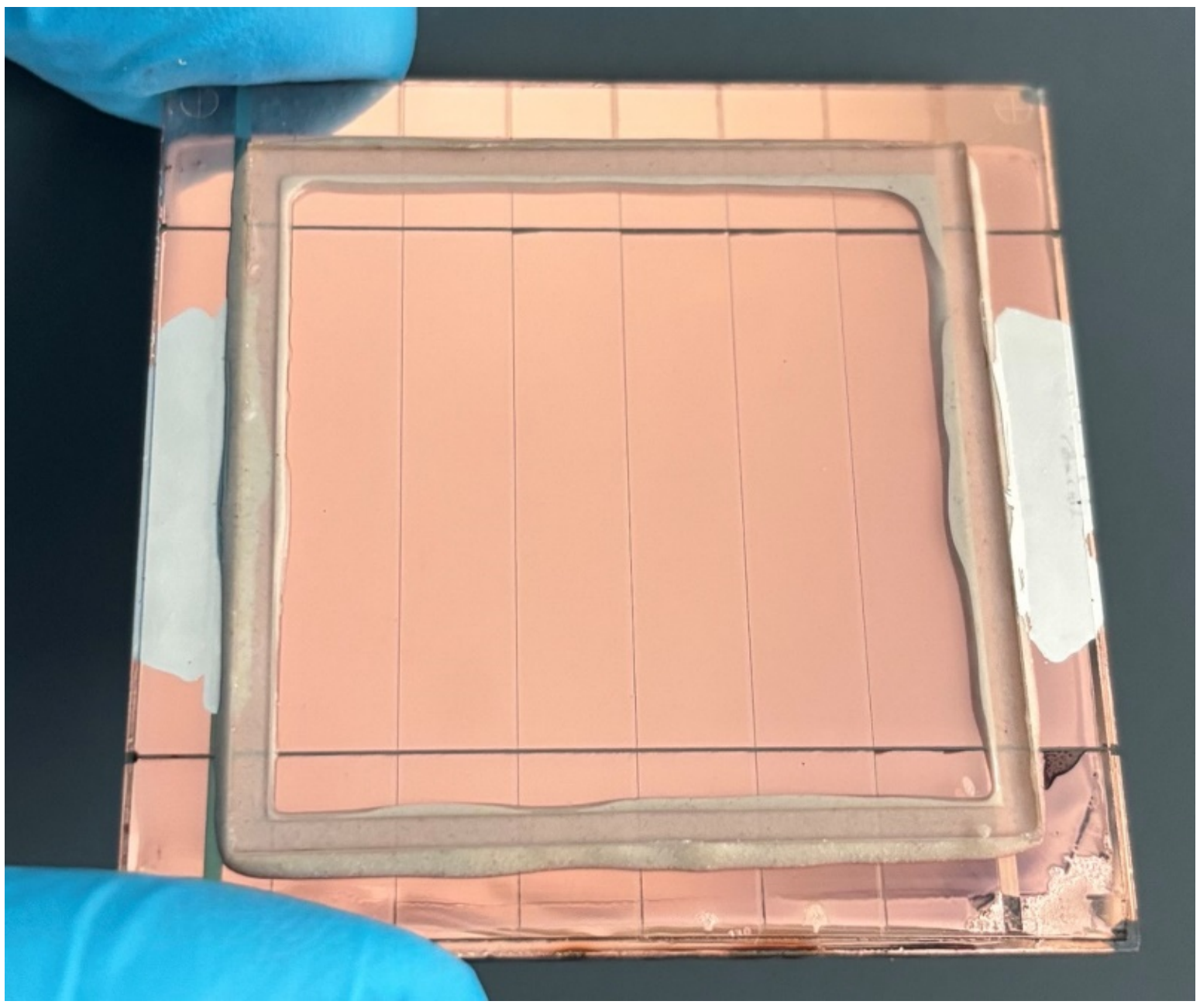

Fig. S22: Photograph of an encapsulated 5 × 5 cm^2 flexible mixed Sn-Pb perovskite solar mini-module.

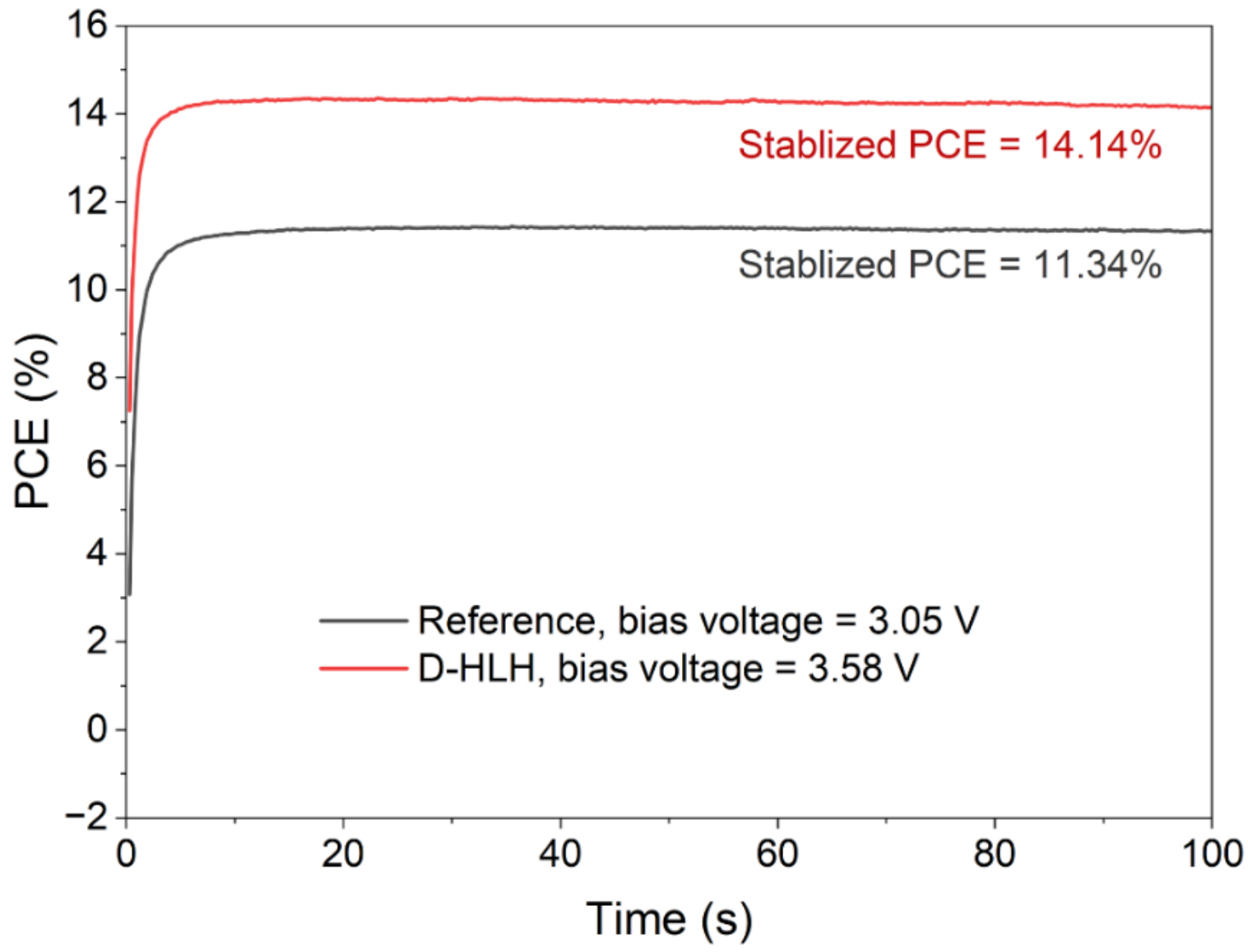

Fig. S23: Stabilized power output of flexible mixed Sn-Pb perovskite solar mini-modules at fixed bias voltage.

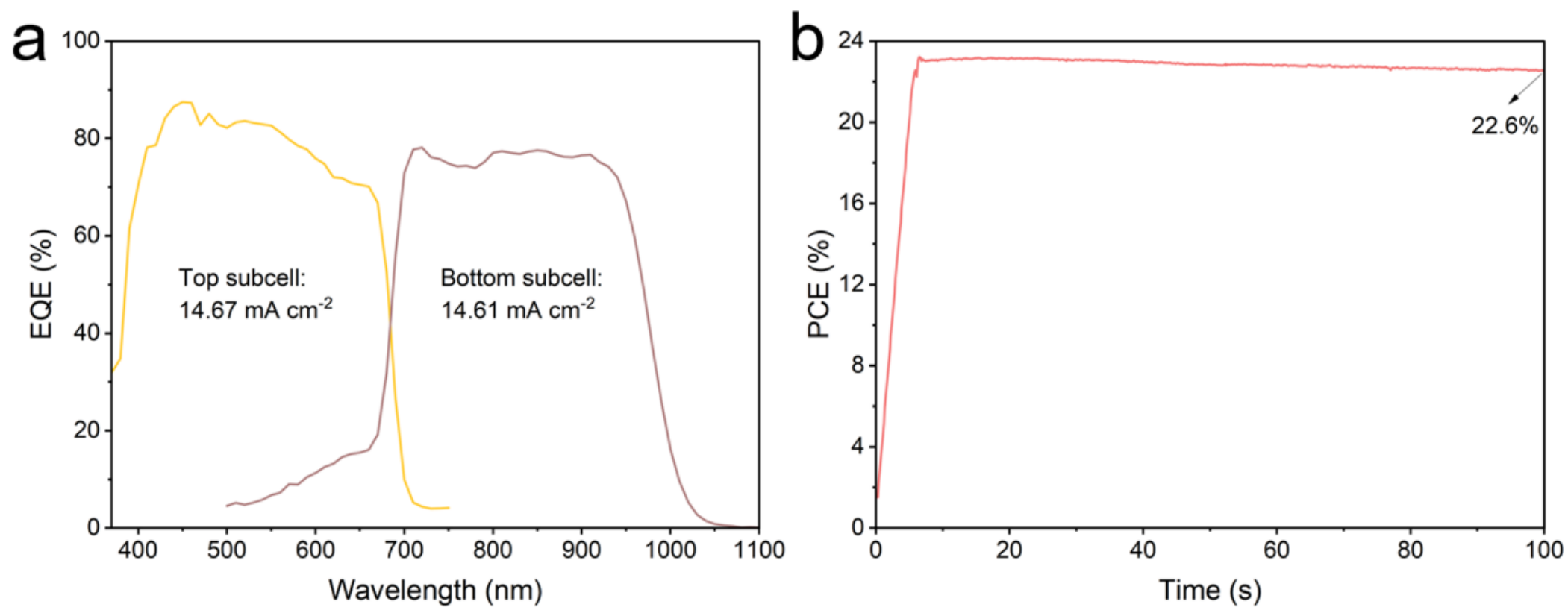

Fig. S24: **(a)** EQE spectra and **(b)** stabilized power output of the best-performing flexible all-perovskite tandem cell.

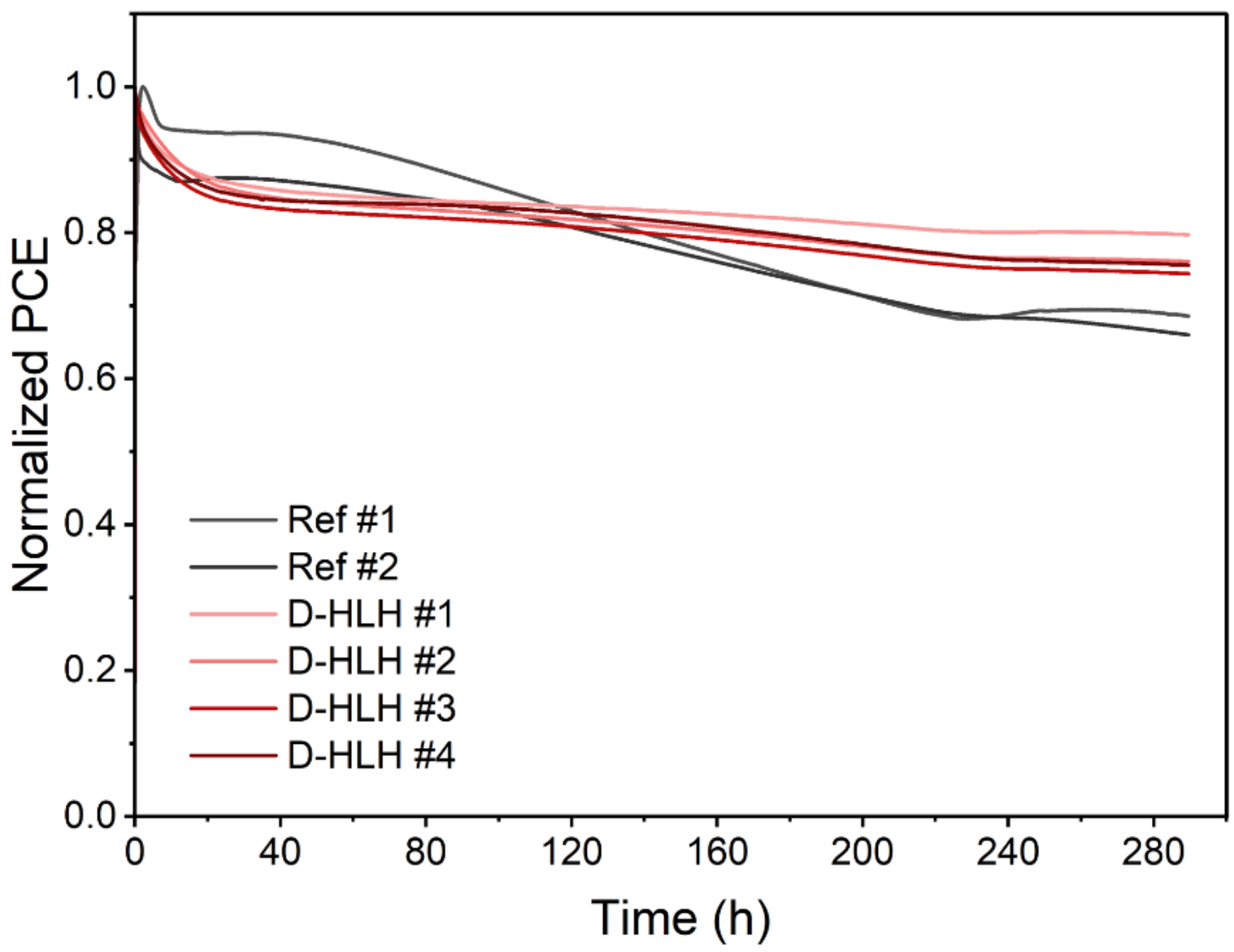

Fig. S25: Operational stability of reference and D-HLH flexible all-perovskite tandem solar cells and minimodules under MPP tracking at ∼55 °C.

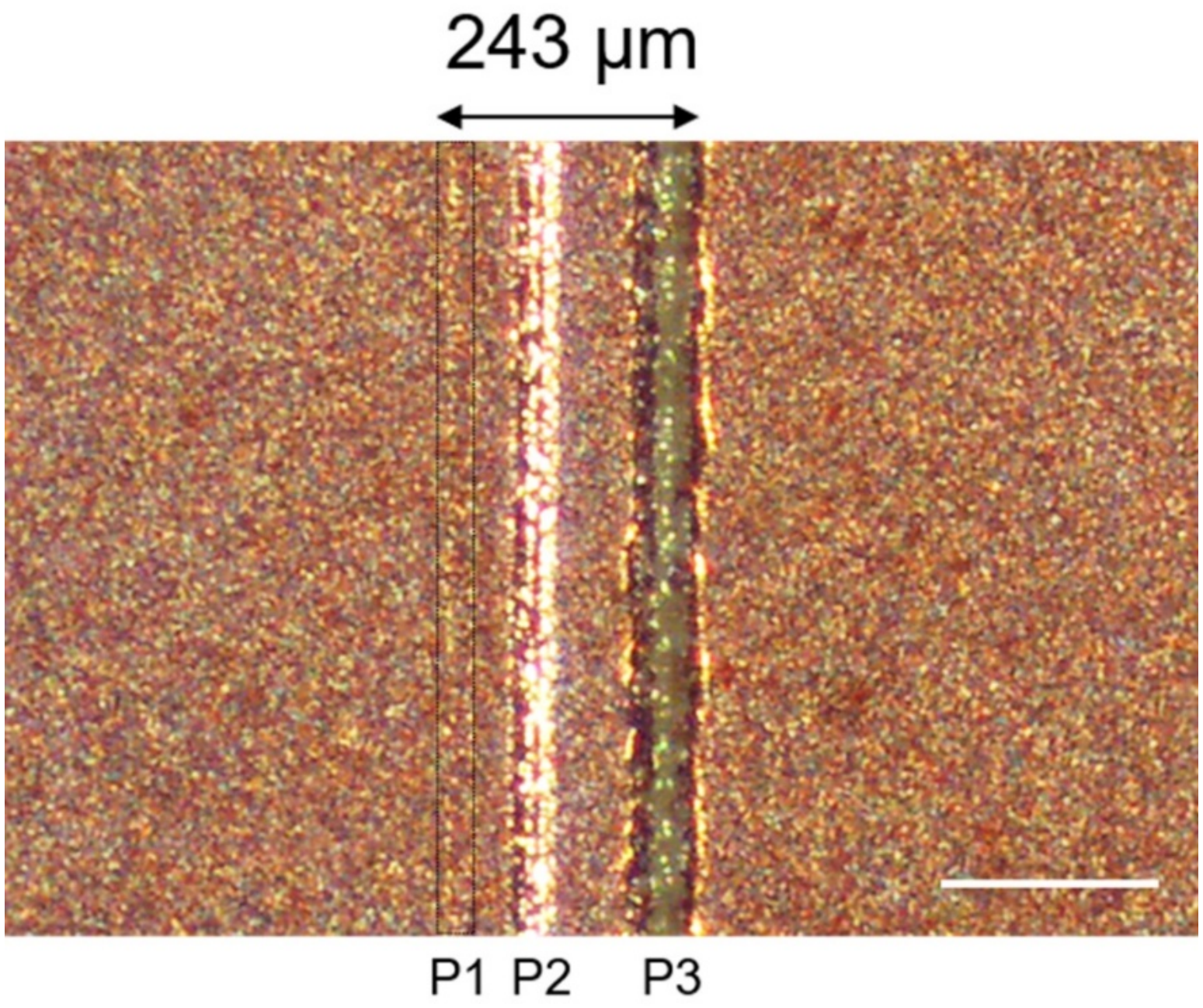

Fig. S26: Optical microscope image of best-performing flexible all-perovskite tandem solar mini-module near dead area. Scale bar: 200 μm.

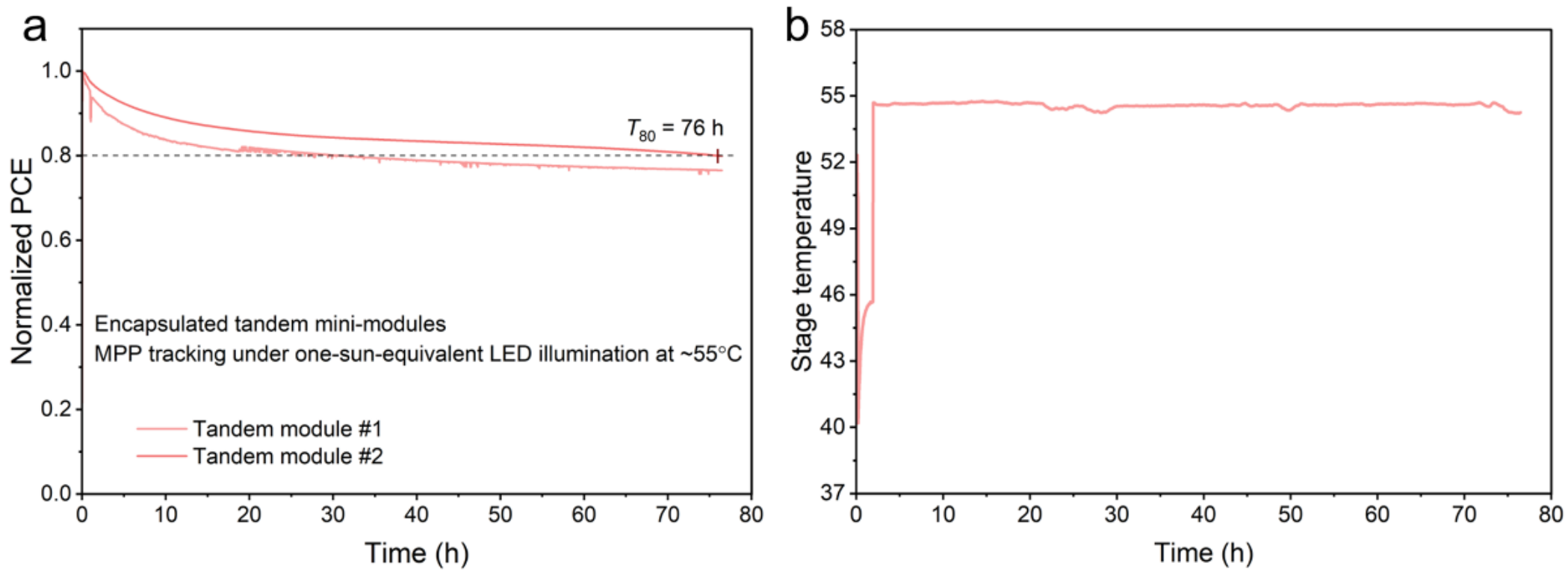

Fig. S27: **(a)** Operational stability of flexible all-perovskite tandem solar mini-modules under MPP tracking and continuous one-sun-equivalent LED illumination. **(b)** Real-time temperature evolution over time during stability test.

Table S1: Biexponential fitting parameters of TRPL decay curves shown in **Fig. 2h**.

	A_1	τ_1	A_2	τ_2	τ_{ave}
Reference	0.64	2.89	0.36	12.32	9.51
D-HLH	0.57	6.27	0.43	25.05	20.33

The TRPL decay curves were fitted using the biexponential function: $I = M_1 \exp\left(\frac{t}{\tau_1}\right) + M_2 \exp\left(\frac{t}{\tau_2}\right) + I_0$, where t is the decay time, I is the PL intensity, I_0 is the background intensity, and M_1 and M_2 are the corresponding pre-exponential amplitudes. The parameters τ_1 and τ_2 represent the fast and slow decay lifetimes, respectively.

Average carrier lifetime (τ_{ave}) is calculated by formular: $\tau_{ave} = \frac{A_1\tau_1^2 + A_2\tau_2^2}{A_1\tau_1 + A_2\tau_2}$, in which $A_1 = \frac{M_1}{I\text{-}I_0}$, $A_2 = \frac{M_2}{I\text{-}I_0}$.

Table S2: Photovoltaic parameters of typical flexible mixed Sn-Pb PSCs at different D-HLH concentrations as shown in Fig. S15.

D-HLH concentration	V_{OC} (V)	J_{SC} from J-V measurement (mA cm^{-2})	FF (%)	PCE (%)	J_{SC} from EQE measurement (mA cm^{-2})
0 mol%	0.712	26.75	71.8	13.7	26.39
0.5 mol%	0.729	27.08	74.2	14.6	26.96
1 mol%	0.749	28.10	75.4	15.9	28.01
1.5 mol%	0.752	27.37	74.9	15.4	27.61

Table S3: Summary of photovoltaic parameters of the best-performing flexible mixed Sn-Pb perovskite solar cell and mini-module and flexible all-perovskite tandem solar cell and mini-module.

Type of devices		Scan direction	V_{OC} (V)	J_{SC} (mA cm^{-2})	FF (%)	PCE (%)
Mixed Sn-Pb perovskite	Cell	Reverse	0.82	28.49	79.0	18.4
		Forward	0.82	28.47	77.8	18.1
	Module	Reverse	4.76	4.46	71.2	15.1
All-perovskite tandem	Cell	Reverse	2.02	14.77	79.1	23.6
		Forward	2.02	14.76	78.1	23.3
	Module	Reverse	12.03	2.29	74.9	20.6
